# Design, characterization and structure–function analysis of novel antimicrobial peptides based on the N-terminal CATH-2 fragment

**DOI:** 10.1038/s41598-022-16303-2

**Published:** 2022-07-14

**Authors:** Pratibha Sharma, Sheetal Sharma, Shubhi Joshi, Panchali Barman, Aashish Bhatt, Mayank Maan, Neha Singla, Praveen Rishi, Md. Ehesan Ali, Simran Preet, Avneet Saini

**Affiliations:** 1 Department of Biophysics, Panjab University, Chandigarh, UT 160014 India; 2 Energy Research Centre, Panjab University, Chandigarh, UT 160014 India; 3 Institute of Forensic Science and Criminology (UIEAST), Panjab University, Chandigarh, 160014 India; 4 Department of Microbiology, Panjab University, Chandigarh, UT 160014 India; 5 Institute of Nano Science and Technology, Sector-81, Knowledge City, Sahibzada Ajit Singh Nagar, Punjab 140306 India

**Keywords:** Biophysics, Computational biology and bioinformatics, Microbiology

## Abstract

The emergence of multidrug resistance coupled with shrinking antibiotic pipelines has increased the demand of antimicrobials with novel mechanisms of action. Therefore, researchers across the globe are striving to develop new antimicrobial substances to alleviate the pressure on conventional antibiotic therapies. Host-Defence Peptides (HDPs) and their derivatives are emerging as effective therapeutic agents against microbial resistance. In this study, five analogs (DP1-5) of the N-terminal (N-15) fragment of CATH-2 were designed based on the delicate balance between various physicochemical properties such as charge, aliphatic character, amphipathicity and hydrophobicity. By means of in-silico and in-vitro studies a novel peptide (DP1) with the sequence “RFGRFLRKILRFLKK” was found to be more effective and less toxic than the N-terminal CATH-2 peptide. Circular dichroism spectroscopy and differential scanning calorimetry were applied for structural insights. Antimicrobial, haemolytic, and cytotoxic activities were also assessed. The resulting peptide was characterized by low cytotoxicity, low haemolytic activity, and efficient anti-microbial activity. Structurally, it displayed strong helical properties irrespective of the solvent environment and was stable in membrane-mimicking environments. Taken together, the data suggests that DP1 can be explored as a promising therapeutic agent with possible clinical applications.

## Introduction

Peptides started establishing their therapeutic niche from humble beginnings as substances isolated from glands such as the isolation and discovery of insulin for the treatment of type I diabetes in the nineteenth century and continued to expand their existence in the pharmaceutical industry^1^. They exhibit wide array of biological roles as cell adhesion motifs, structural peptides, cell-penetrating and tumor homing peptides, antimicrobial peptides, peptide hormones, growth factors and matrix metalloprotease substrates, amyloid peptides, neuropeptides, and other miscellaneous natural peptides and peptide tags^2^. Peptides lying in the sweet spot between small-molecule and protein therapeutics are likely to draw increasing inclination to the research and development of peptide therapeutics, which in turn could lead to their substantial increase in the clinical pipeline.

In the present clinical settings, when conventional antibiotics are becoming ineffective against many microbes, antimicrobial peptides (AMPs), are emerging as novel antimicrobials agents^3^. They have been exhibiting potent broad-spectrum antimicrobial and/or immunomodulatory properties for millions of years and have continuously fought the evolution of bacterial resistance^4^. AMPs are short, positively charged peptides and act as the first line of defence against microbial invasion as they are ancient weapons of innate immunity and widely distributed throughout the life kingdom^5^. Although many potent AMPs have been reported and some of them are in clinical phase 1 and phase 2 trials for topical applications as skin infections, chronic leg ulcers, wound infections and ear and eye infections^6–8^, but toxicity against eukaryotic cells, poor efficiency, and lack of knowledge about the mechanisms of action are key obstacles for their clinical application^9^.

The binding of AMPs to bacterial membranes is influenced by the net charge and hydrophobicity of the AMPs. Gram-negative bacteria contains a thin coating of peptidoglycan on their outer membrane, with lipopolysaccharide (LPS)^10^. Gram-positive bacteria, on the other hand, have a thick coating of peptidoglycan around the cytoplasmic membrane, with lipoteichoic acid running through it. These virulence factors, the capsular polysaccharides (CPS) and complete polysaccharides (LPS) provide pathogenic potential to the bacteria^11^. Although, both CPS and LPS are often found associated together, they differ in their structural composition and play an important role in infection pathogenesis. LPS consists of lipid A, a core oligosaccharide region, and a serotype specific O-antigen, while the CPS is composed of a K-antigen, which is a polymer that forms the capsule^12^. The K antigens of *Escherichia coli* (*E.* *coli*) are further divided into Group I and II, with the group I containing hexuronic acid as acidic components and usually co-expressed with O antigens^13^. The group II K antigens may contain hexuronic acids, N-acetylneuraminic acid (NeuNAc), or d 2-keto-3-deoxymanno-octonic acid (KDO) as acid components, and have a higher charge density compared to Group I antigens. The different groups of CPS further vary in their structural composition as well as sites of attachment. The capsule of CPS and O-antigen of LPS provide protection to the bacterium against bactericidal activity of host-defence^11^.

The positively charged AMPs interact with the negatively charged cell wall components of bacterium, such as LPS and lipoteichoic acid (LTA) via electrostatic interactions, resulting in membrane instability and permeabilization. The hydrophobic residues then allow the AMPs to further enter into the bacterial membrane's bilayer leading to membrane lysis due to a high cytoplasmic osmotic pressure and membrane’ integrity is compromised^14^. The membrane-active AMPs act by generating pores in the bacterial membrane, which gives rise to four types of AMP’s mechanism of action, the barrel-stave, carpet, toroidal, and detergent-like models, based upon the amino acid residues, hydrophobicity, charge and length of the peptide. According to the barrel-stave model, the AMPs perpendicularly enter into the membrane's lipid bilayer and form a channel. In the carpet model, the AMPs blanket the membrane's surface without producing distinct holes. The AMPs create a channel by inserting perpendicularly into the lipid bilayer without particular peptide–peptide interactions in the toroidal pore model, and, in the detergent-like model, the AMPs split membranes into minute fragments in the same way as a detergent does^14,15^.

In this study we report the design and structure–function characterization of a novel peptide “RFGRFLRKILRFLKK” that is an analog of the N-terminal fragment of chicken cathelicidin-2 (N-15 CATH-2). CATH-2 belongs to a major family of host-defence peptides called as cathelicidins^16^ and is an arginine-lysine rich peptide with both immunomodulatory and strong broad-spectrum antibacterial activity, but with a noticeable toxicity to mammalian cells^17^. Moreover, being the truncated variant of the native peptide, it can be actively explored for the desired activity and at the same time minimizing the cost for peptide synthesis. Various studies have revealed that the potency of such peptides depends on interrelated structural and physiochemical properties like hydrophobicity, cationicity and amphipathicity^18^. This study focuses on in-silico designing of peptide analogs with enhanced antimicrobial activity and low cytotoxicity based on the parameters mentioned above. A thorough physiochemical analysis of the designed peptide analogs and Molecular Dynamics (MD) studies were performed on the N-terminal fragment of chicken cathelicidin-2 (CATH-2) as well as the designed peptide analogs in different environments. The peptide analogs were selected for further in-vitro studies based on their structure and structure–function relationship studies. The secondary structure of the peptides was validated using Circular Dichroism (CD) spectroscopy and antimicrobial activity was evaluated by determining the minimum inhibitory concentration (MIC) against Gram-positive and Gram-negative bacterial strains*.* The haemolytic activity, and cytotoxicity studies were also carried out. The therapeutic index (TI) was calculated to assess the cell selectivity of the peptides. The results helped us to explore the role of the various physiochemical properties in peptide structure and mechanism of action. Hence, these findings could represent a significant addition to issues related to rationalization for antimicrobial peptide designing and optimization.

## Results and discussion

### Peptide designing

Antimicrobial peptides tend to adopt amphipathic structures with hydrophilic (positively charged) and hydrophobic faces. This facilitates their interaction with negatively charged microbial membranes, their insertion and hence their antimicrobial potency^19^. Further, numerous reports on AMPs have suggested that helicity increases the amphipathicity of a peptide, which aids in microbial membrane disruption^20–22^.

Structure–activity relationship (SAR) studies have revealed how the physiochemical properties play a crucial role in the antimicrobial activities of these peptides and how changing these parameters can act as a strategy to design novel antimicrobial peptides with increased efficacy^23^. The two faces of the amphipathic peptide, the hydrophobic face, which helps in membrane penetration of the peptide in the cell; and the charged face, which helps in membrane disruption, arise due to the helical structure of the peptide^24^.

**Figure 1:**
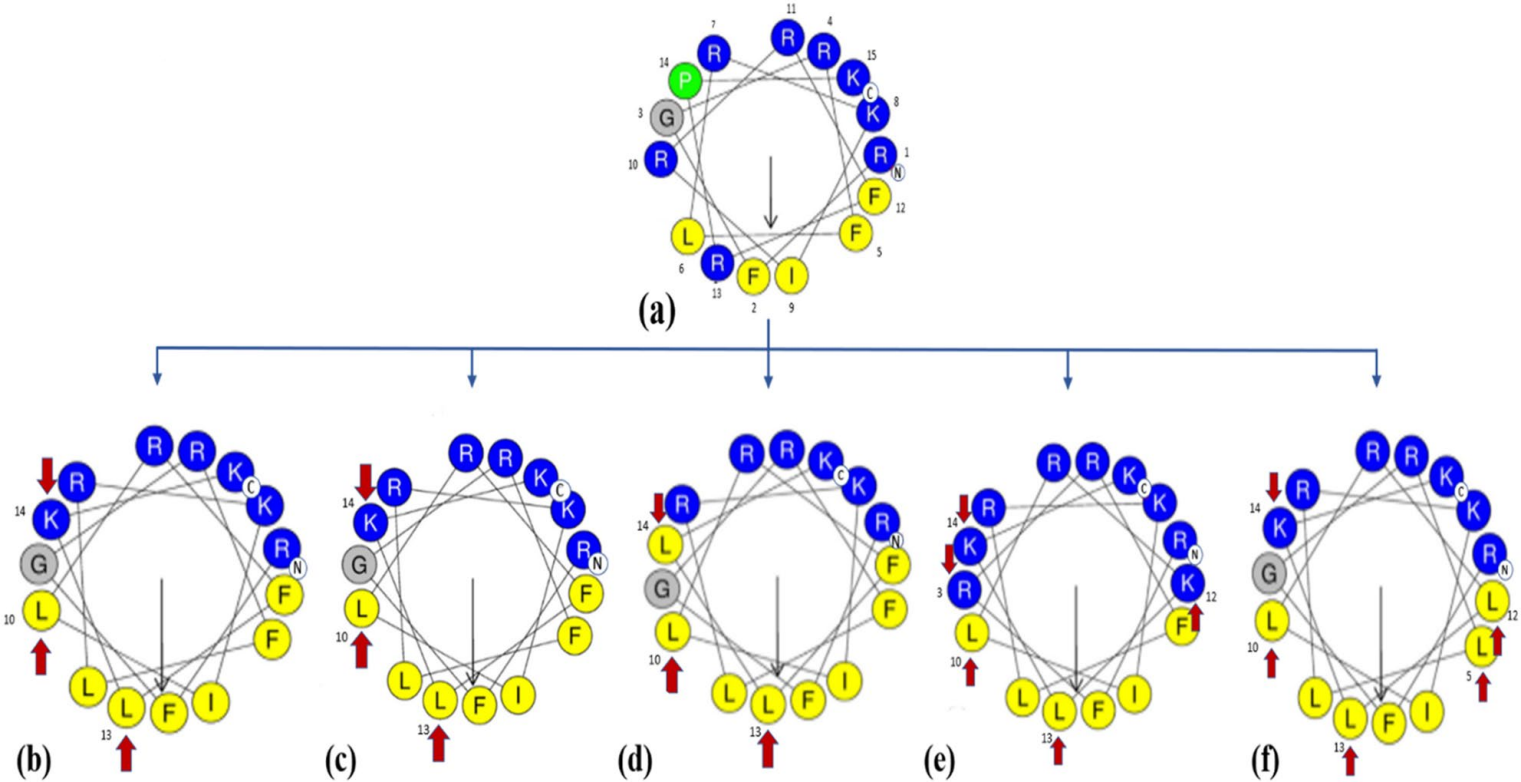
Helical wheel projections of N-15 CATH-2 (**a**), DP1 (**b**), DP2 (**c**), DP3 (**d**) DP4 (**e**) and DP5 (**f**). Residues are color-coded with non-polar hydrophobic residues in yellow, polar basic residues in dark blue, glycine in grey, and proline in green circles. The black arrow in the helical wheel corresponds to the hydrophobic moment and the red arrow indicates the residues substituted in N-15 CATH-2.

The physiochemical parameters of the N-15 CATH-2 were analysed (Table 1) using ProtParam (ExPASy Proteomics Server: https://web.expasy.org/protparam.html). Also, the mean hydrophobicity (H), and the helical wheel projections were obtained using the Heliquest software (http://heliquest.ipmc.cnrs.fr.html)^25^. As evident from the helical wheel projection of N-15 CATH-2 peptide (Fig. 1a) the amino acids—R^10^, R^13^ and P^14^ were disrupting the hydrophobic and hydrophilic faces of the amphipathic structure and hence were selected for substitution. All the designed peptide analogs as shown Fig. 1, were hence designed by replacing/substituting these outliers with the aim to attain an amphipathic helical structure while maintaining the delicate balance between charge-aliphaticity-hydrophobicity (Table 1). In DP1, R^10^, R^13^ and P^14^ were replaced with L^10^, L^13^ and K^14^ so that distinct hydrophobic and hydrophilic faces of the amphipathic helical structure could be achieved (Fig. 1b). The peptide had a net positive charge of + 7 and a negative GRAVY (Grand Average of Hydropathy) value that implied that it is hydrophilic in nature (Table 1). DP2 was similar to DP1 except the 14^th^ amino acid position (Fig. 1c). As both lysine and arginine are frequently found in antimicrobial peptides so in DP2 and DP3, L^10^, L^13^ and R^14^ and L^10^, L^13^ and L^14^ substitutions were made, respectively (Fig. 1d) which led to a larger hydrophobic face and a slightly smaller hydrophilic face with a net positive charge of + 6 in DP3. Contrary to this, DP4 was designed with a smaller hydrophobic face and a larger hydrophilic face (Fig. 1e). In DP5 the ideal amphipathic structure was retained but we played around the aliphatic character of the peptide by making L^5^, L^10^, L^12^, L^13^, K^14^ substitutions (Fig. 1f).

**Table 1:** Primary sequences and physiochemical properties of N-15 CATH-2 and its designed peptide analogs.

Peptide	Sequence	Charge	Aliphatic index	Isoelectric pH	GRAVY	Hydrophobicity (H)
N-15 Cath-2	RFGRFLRKIRRFRPK	+ 8	52.0	12.7	− 1.34	0.103
DP1	RFGRFLRKI**L**RF**LK**K	+ 7	104.0	12.5	− 0.387	0.351
DP2	RFGRFLRKI**L**RF**LR**K	+ 7	104.0	12.6	− 0.427	0.349
DP3	RFGRFLRKI**L**RF**LL**K	+ 6	130.0	12.5	0.127	0.530
DP4	RF**R**RFLRKI**L**R**KLK**K	+ 9	104.0	12.6	− 1.107	0.098
DP5	RFGR**L**LRKI**L**R**LLK**K	+ 7	156	12.5	− 0.253	0.339

### Molecular dynamics simulations

Structural preferences of peptides or proteins are highly sensitive to the local environmental conditions that refer to the solvation interface which communicates bulk properties of the solvent (like temperature, pressure, dielectric constant, etc.) to the peptide. Therefore, molecular dynamics simulations with three different starting conformations i.e., Conformation-A (Φ = 180°, ψ = 180°), B (φ = − 57°, ψ = − 47°) and C (φ = − 139°, ψ = 135°) of the N-15 CATH-2 peptide and all the designed peptide, DP analogs were carried out using the GROMACS software^26^ in two different solvents-water and DMSO under NVT conditions at 300 K. The conformational results in terms of Φ, ψ values of the average structures of the peptides built over the last 500 ps of the 20 ns MD simulation run in both the solvents were obtained (Supplementary Table S1–S6) and analysed. Ramachandran plots and molecular views showing the stereo chemically allowed combinations of the Φ, ψ values for the various peptide analogs in water and DMSO are as shown in Supplementary Figs. S1–S3.

MD simulation studies revealed that the N-15 CATH-2 peptide adopted a β-strand type structure in water with average Φ, ψ values of − 93.7°, 121.6°, except the residues Leu (6) and Arg (7,11) (Supplementary Figs. S1a and S2a). The deviations from the uniform secondary structure are attributed to the formation of a type-I β-turn like structure by Arg (7) and Lys (8) that adopt Φ, ψ values of − 73.0°, − 48.0° and − 85.0°, 99.7° respectively. In general, type-I β-turn is characterized by torsion angles of {− 60°, − 30°} for (i + 2)th and {− 90°, 0°} for (i + 3)th residue^27^. This type of turn has been referred to as “open turn” in literature and is characterized by absence of hydrogen bond and the Φ, ψ angles tend to deviate ± 30° from the ideal values^28,29^. Moreover β-turns are also characterized by the distance between C_α_ (i) and C_α_ (i + 4) which must be smaller than 7 Ȧ^30^ and the distance in case of this turn is 5.9 Ȧ.

In DMSO, the peptide was again found to be stable in β-strand type secondary structure that spanned the entire length of the peptide (Supplementary Fig. S1b and S2b). It is interesting to note that in DMSO another conformation with helical secondary structure across residues 3–11 with average Φ, ψ values of − 59.0°, − 45.5° was only 13 kcal/mol less in stability. This energy difference is not much and may allow population of both β-strand type and helical conformation under specific environmental conditions. The β-strand structure of N-15 CATH-2 was stabilized in the presence of DMSO due to the formation of H-bonds between the sulfoxide group and peptide backbone^31–33^.

MD simulations of the designed peptides revealed that the energetically most stable conformation of the peptide DP1 adopted a helical secondary structure except the terminal residues. This structure is stabilized by the formation of 7 strong intra-peptide H-bonds between the ith and ith + 4 residues (Supplementary Fig. S1c and S2c). Even in DMSO, the helical secondary structure was observed as the most stable structure with the presence of 9 strong intra-peptide H-bonds between the ith and ith + 4 residues (Supplementary Fig. S1d and S2d) contributing towards the stability of the peptide in DMSO.

DP2 also adopted a helical structure in water that was slightly destabilized in DMSO by the formation of a random coil like structure at the C-terminal (Supplementary Fig. S3a). This may be attributed to the steric hindrance caused by the bulky guanidinium side chain of arginine (R14) as compared to lysine (K14) in DP1.

A random coil structure with absence of any uniform secondary structure was observed for the peptide DP3 in water with higher hydrophobicity than DP1 and DP2 (Supplementary Fig. S3b). Changing the solvent to DMSO stabilized DP3 in β-strand type secondary structure. Further, MD simulations revealed that peptides DP4 and DP5 too adopted a β-strand type structure and here this secondary structure was observed irrespective of the solvent being water or DMSO (Supplementary Fig. S3c,d). Increasing the charge beyond + 7 (N-15 CATH-2 and DP4) or increasing the aliphatic character (DP5) favoured β-strand type secondary structure, whereas helical structures were more stable when the charge was + 7 along with a balance between aliphatic character and hydrophobicity (DP1, DP2). Thus, to understand the role of alpha helical secondary structure, DP1 was further simulated to 100 ns in water and in peptide-micelle systems.

### MD simulations of peptide-micelle systems

**Figure 2:**
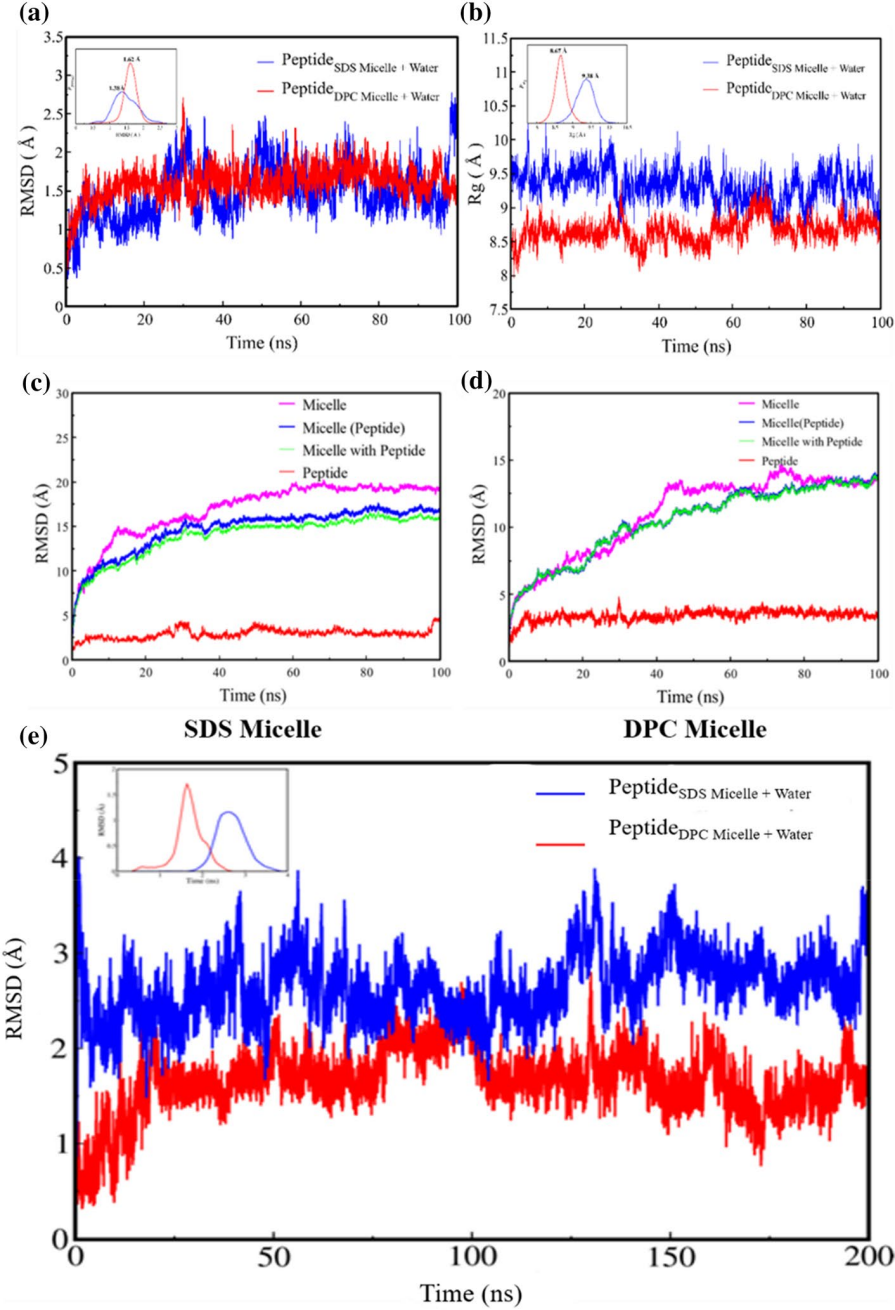
Conformational (RMSD) movement (**a**), and radius of gyration of the peptide, DP1 in SDS-water micelle complex and DPC-water micelle complex (**b**). Conformational (RMSD) movement of SDS (c) and DPC micelle (**d**) in the absence, presence, and the micelle complex with the peptide. Peptide backbone RMSD plotted for the entire 200 ns production dynamics (**e**).

The surface electrostatic potential of DP1 was calculated through PDB2PQR server (Supplementary Fig. S4). This surface charge positivity may be responsible for interactions with the head groups of the micelle. Further, 100 ns molecular dynamics simulations were performed for this peptide in water, peptide in SDS-water micelle complex and peptide in DPC-water micelle complex. As evident from the MD simulations the energetically most stable conformation of the peptide, DP1 was seen to adopt a helical secondary structure in water as well as DMSO due to the presence of strong intra-peptide bonds. Therefore, the helical secondary structure was chosen as starting conformation for MD simulations in micelle systems. The anionic SDS micelles that mimic the prokaryotic membrane environment and zwitterionic DPC micelles that mimic the eukaryotic membrane environment were applied^34^. Root Mean Square Distance (RMSD) and radius of gyration were analyzed. RMSD analysis (Fig. 2a) revealed a stable conformation of the peptide present in both the micelles, it does not deviate significantly from the reference structure. The most probable distribution values of RMSD are1.38 Å for the Peptide_SDS+Water_ and 1.68 Å for Peptide_DPC + Water_. In the absence of micelle, DP1 peptide was free to move in the simulation box fill with water (DP1 Peptide_Water_ system) and simulated for 200 ns long trajectory (Fig. 2e) after the formal 5 ns equilibrations steps, indicating the thermal equilibrations, however, experiencing larger conformation fluctuations during the dynamics. Figure 2 considers the initial 100 ns (i.e., 105 ns in total) as part of the equilibration. In comparison to other systems, DP1 peptide in water showed large fluctuations (Supplementary Fig. S5a) whereas, constant radius of gyration showed a compact structure (Fig. 2b).

**Figure 3:**
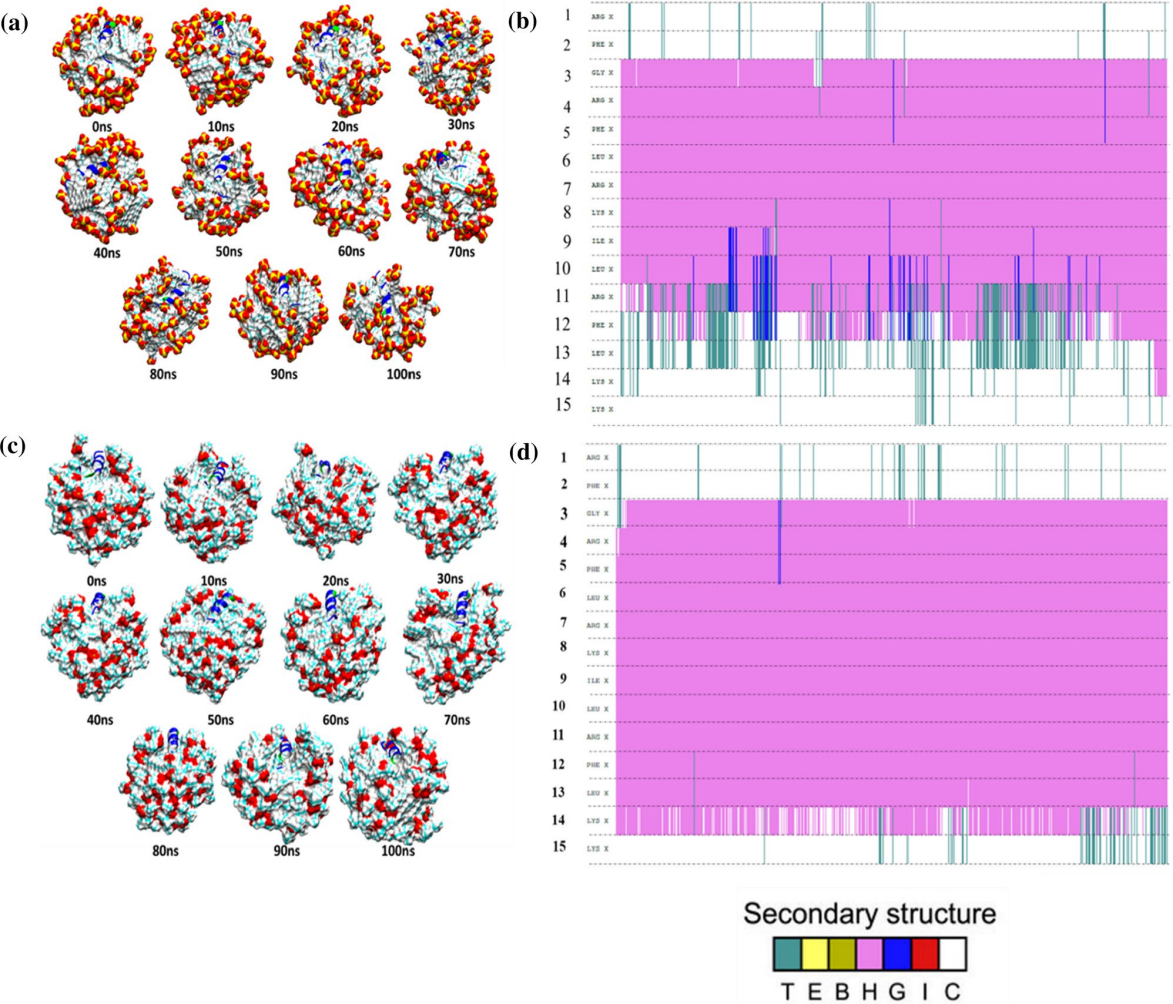
Selected snapshots of the SDS micelle with peptide (**a**) and DPC micelle with peptide complex (**c**). The secondary structure analysis of the peptide in SDS micelle (**b**) and peptide in DPC micelle with water complex (**d**) throughout the 100 ns trajectory. y-axis represents the amino acid sequence of DP1 and the colored area represents the secondary structure it adopts. (Turn, T (green); helix, H (pink); 310 helix, G (blue)).

From careful investigation of secondary structure, it is quite evident that DP1 peptide always resides in the hydrophobic chain of both the micelles (Fig. 3a,c). Looking into the DP1 peptide secondary structure on the DPC micelle system, it showed strong α-helix (H) content whereas, in the SDS micelle complex peptide system 3_10_ helix (G) and turn (T) structure are present (Fig. 3b,d). So, DP1 peptide might be affecting the micelle structure and have a loss of secondary structure content due to the peptide itself. In the peptide-water system, the secondary structure of DP1 peptide was found to fluctuate from the ideal alpha helix to 3_10_ helix. Peptide changes its conformations from α-helix (H) to 3_10_ helix (G), turn (T) and vice versa during the dynamics (Supplementary Fig. S6a,b).

The Hydrogen bond (H-bond) interactions between DP1 peptide and selected atoms (oxygen and sulphur) of SDS micelles were also assessed (Supplementary Fig. S6a). N-terminal Arg1 amino acid (aa) residue of DP1 peptide interacts with the micelle with the highest occurrences of interactions (1–7) with the SDS micelle polar group. Phe2, Gly3 and Arg4 also showed H-bond interactions with the polar group atoms. Hydrophobic residue 5, 6, 10, 12 and 13 displayed minimal H-bond interactions which might be due to the dependence upon the orientation of the micelle surface. Hydrophobic residues tend to interact with the hydrophobic chain of the micelle. Due to this behavior Ile9 aa residue permanently resides on the hydrophobic chain of the micelle. The distance between Centre of Mass (COM) of each residue and the hydrophobic core of the SDS micelle was also calculated (Supplementary Fig. S7b). The distance of the hydrophobic residues from the hydrophobic core was less than 15 Å except for glycine. These observations revealed that only hydrophobic amino acids were able to interact with the micelle core. N-terminal residues of the DP1 peptide have been seen to form H-bond interactions with the polar groups of DPC micelle. Here, hydrophobic amino acids interact with the hydrophobic chain of the DPC micelle. Similar interactions were seen between N-terminal residues and DPC micelle (Supplementary Fig. S7a). The distance between COM of each residue and the hydrophobic core of the DPC micelle was calculated (Supplementary Fig. S7b). The distance of the hydrophobic residues from the hydrophobic core was less than 15 Å for Ile9 and Lys13.

The conformational movement of the SDS (Fig. 2c) and DPC (Fig. 2d) micelles was calculated in terms of RMSD. These observations revealed that the RMSD for both the micelles was less in the presence of the DP1 peptide in the simulation cell as compared to the micelles with ions only. Also, DPC micelle with peptide showed less fluctuations as compared to the SDS micelle.

These results revealed that the DP1 peptide always interacts with the active groups of the SDS and DPC micelles through the N-terminal amino acid residue. Hydrophobic amino acids interact with the hydrophobic chains of the micelles and does not get inserted into the micelle core. The secondary structure analysis revealed that the peptide had a fluctuating helical structure in water that was stabilized and more uniformly conserved in both the micelle systems.

### Changes in the organization and phase behavior of different MLVs in the absence and presence of peptides as depicted by differential scanning calorimetry

**Figure 4:**
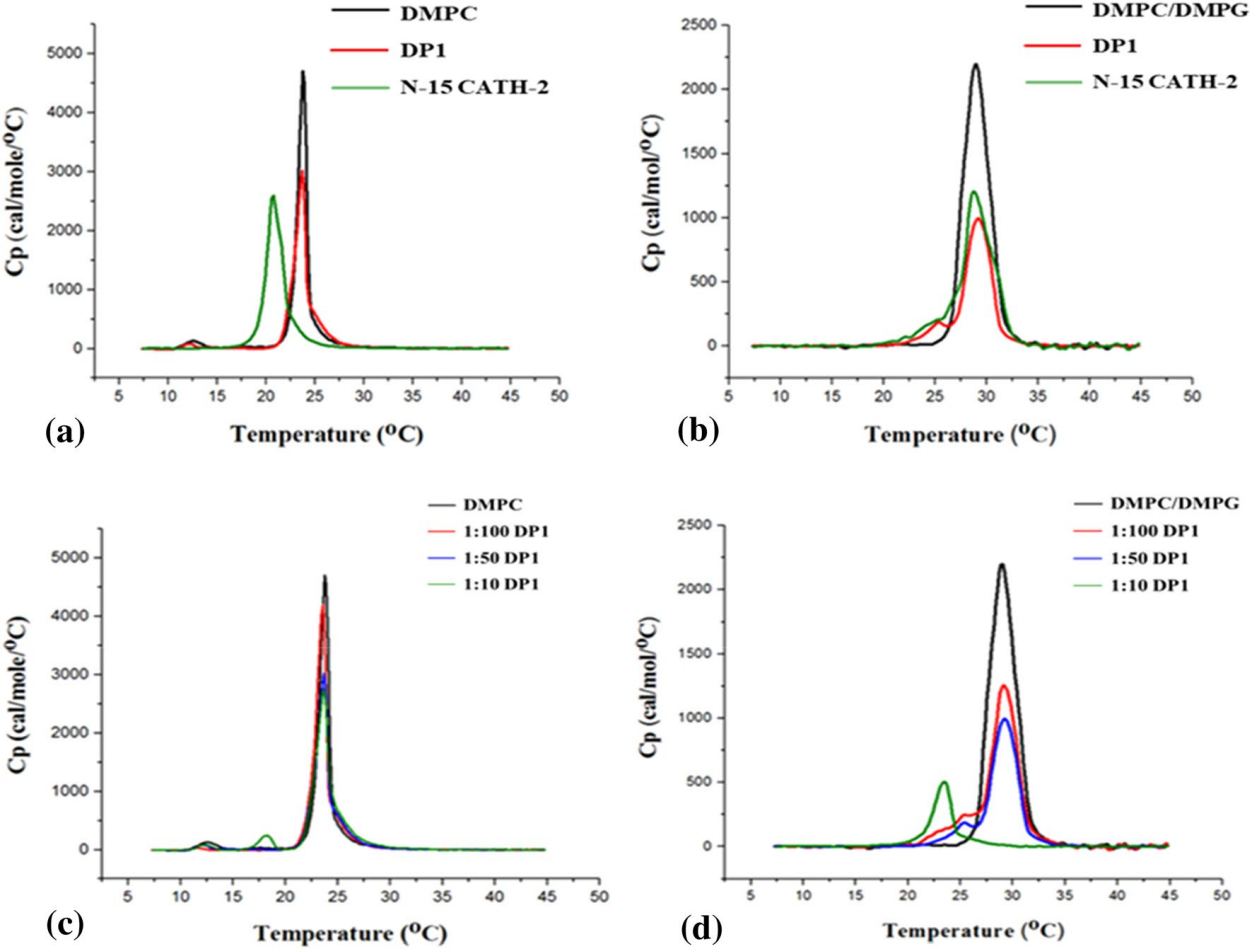
DSC heating thermograms for DMPC (**a**) and DMPC/DMPG (**b**) multilamellar vesicles with and without peptides. Scans were acquired at peptide/lipid molar ratio of 1:50. thermograms of DP1 with DMPC (**c**) and DMPC/DMPG (**d**) multilamellar vesicles at different peptide:lipid molar ratios.

MLV of DMPC exhibited two endothermic events (Fig. 4a) in the temperature range studied with the low-enthalpic, broad pre-transition peak (T_p_) arising from the conversion of the lamellar gel phase (L_β'_) to the ripple gel phase (P_β'_) and was observed at about 13 °C and the more energetic and more cooperative (narrow) main phase or chain-melting transition peak (T_m_) arising from the conversion of (P_β'_) to the liquid-crystalline phase (L_α_) centered at 23.7 °C. MLVs of DMPC/DMPG (3:1) presented only a single high-enthalpic and broad main phase transition at 29 °C (Fig. 4b). As depicted in Fig. 4, addition of peptides into the lipid solution leads to a significant reduction of the calorimetric enthalpy (ΔH) of the main transition (Supplementary Table S7). The decrease in ΔH values (Supplementary Table S7) is greater in case of negatively charged DMPC/DMPG MLVs; emphasizing the crucial role of electrostatic interactions as one of the initial steps of peptide-membrane interactions, since both the peptides are cationic in nature^5,35^. However, there was no significant change in T_m_ values and cooperativity of the main phase transition as seen in Fig. 4b. In contrast to this, interaction of N-15 CATH-2 with DMPC vesicles lead to decrease in T_m_ value by 2.82 °C (Supplementary Table S7) and disappearance of T_p_ peak indicating that the peptide favors the main transition by intercalating the lipid bilayer and disrupting the packaging of carbon chains. It clearly depicts that N-15 CATH-2 has a fluidizing effect on the membrane^36^.

To further study the effect of different peptide:lipid molar ratios of DP1 on MLVs, DSC thermograms were obtained and studied. The results clearly show that DP1 strongly perturb the structural integrity of DMPC/DMPG vesicles which are anionic in nature. Whereas, it has no significant effect on the zwitterionic DMPC vesicles. As seen in Fig. 4c, upon increasing DP1 concentration to DMPC vesicles, there is a change in the enthalpy of the main transition but no change in the temperature and cooperativity of the main phase transition was observed, indicating that the peptides interacted with the polar head groups and glycerol backbone region of the phospholipids only (Fig. 3, Supplementary Table S7)^37^. However, the binding of increasing quantities of DP1 to negatively charged MLVs strongly reduces the T_m_ by 5.74 °C along with the enthalpy of the main phase transition, indicating interaction between DP1 and the polar head groups and acyl region of the phospholipids, penetration of the peptide into the hydrophobic domain and fluidification of the membrane by disrupting the acyl chain. The main phase transition event depicts trans-gauche isomerization of the lipid acyl chains which alters the acyl chain packing in the DMPC/DMPG lipid vesicles and hence increasing lipid mobility or fluidity.

### Circular dichroism spectroscopy

**Figure 5:**
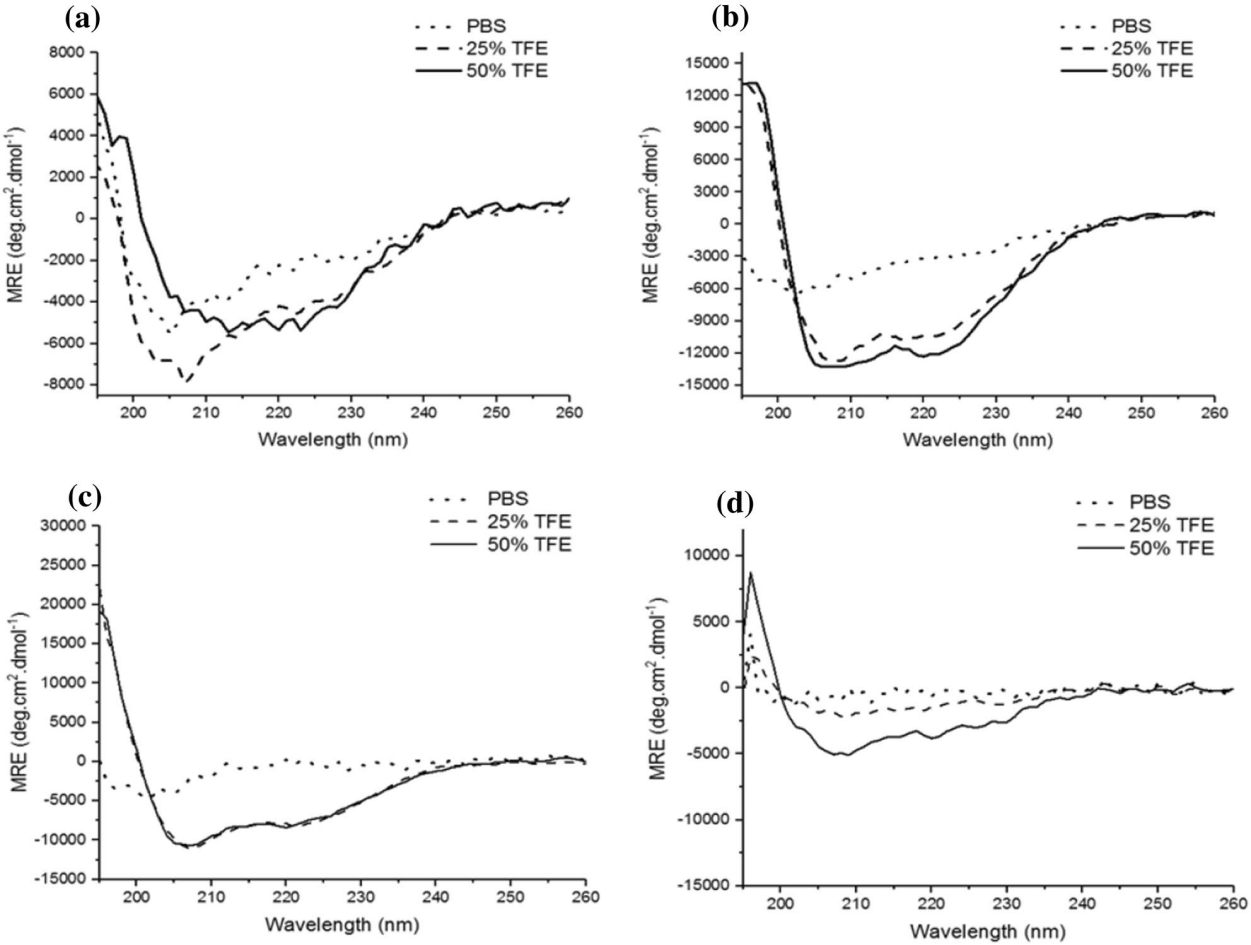
CD spectra exhibited by N-15 CATH-2 (**a**), DP1 (**b**), DP2 (**c**) and DP3 (**d**) in aqueous (…) and TFE-containing buffer (− 25% TFE and − 50% TFE).

Structural conformation of the peptides has been shown to relate to their antimicrobial activity, the secondary structure analysis of peptides was carried out by CD spectroscopy in phosphate buffer saline (PBS), 25% TFE and 50% TFE in buffer. The peptides showed random coil or extended conformation in buffer with a shallow minima at ~ 200 nm and a flat baseline up to 220 nm (Fig. 5)^38^. However, the values of molar ellipticities increased significantly when peptides were transferred to so called membrane-mimetic media TFE which shows that their secondary structure is solvent inducible^39^. The molar ellipticity curve of N-15 CATH-2 in 25% TFE solution reflected a conformational ensemble of type Ι/ΙΙΙ β-turns and unstructured backbone conformations having both positive and negative ellipticity values and broad minima at 210–220 nm (Fig. 5a), characteristic of β-stranded peptides^40^.

In contrast to this, DP1 showed a typical α-helical spectrum in 25% TFE, with double minima around 208 and 222 nm (Fig. 5b) and percentage helicity of 32% calculated using MRE value at 222 nm. The increase in concentration of TFE, further leads to helical stabilization with 37% helical content in 50% TFE solution. However, the N1-15 CATH-2 peptide exhibited poor helical properties in 50% TFE solution, probably due to the presence of proline at 14th position^41^. The CD spectra in case of DP2 is similar to that of DP1 with the propensity to adopt helical structure in TFE, however percentage helicity i.e., 25% is less as compared to that of DP1. The substitution of one of the positively charged residues with leucine lead to disappearance of negative ellipticity at 208 and 222 nm, with random coiled structure in PBS as well as in 25% TFE and poor helical content even in 50% TFE. Based on the MD simulations, secondary structure evaluation, DSC studies and CD results; DP1, DP2, and DP3 along with N-15 CATH-2 were considered for further in-vitro studies.

### Antimicrobial activities against Gram-positive and Gram-negative organisms

**Figure 6:**
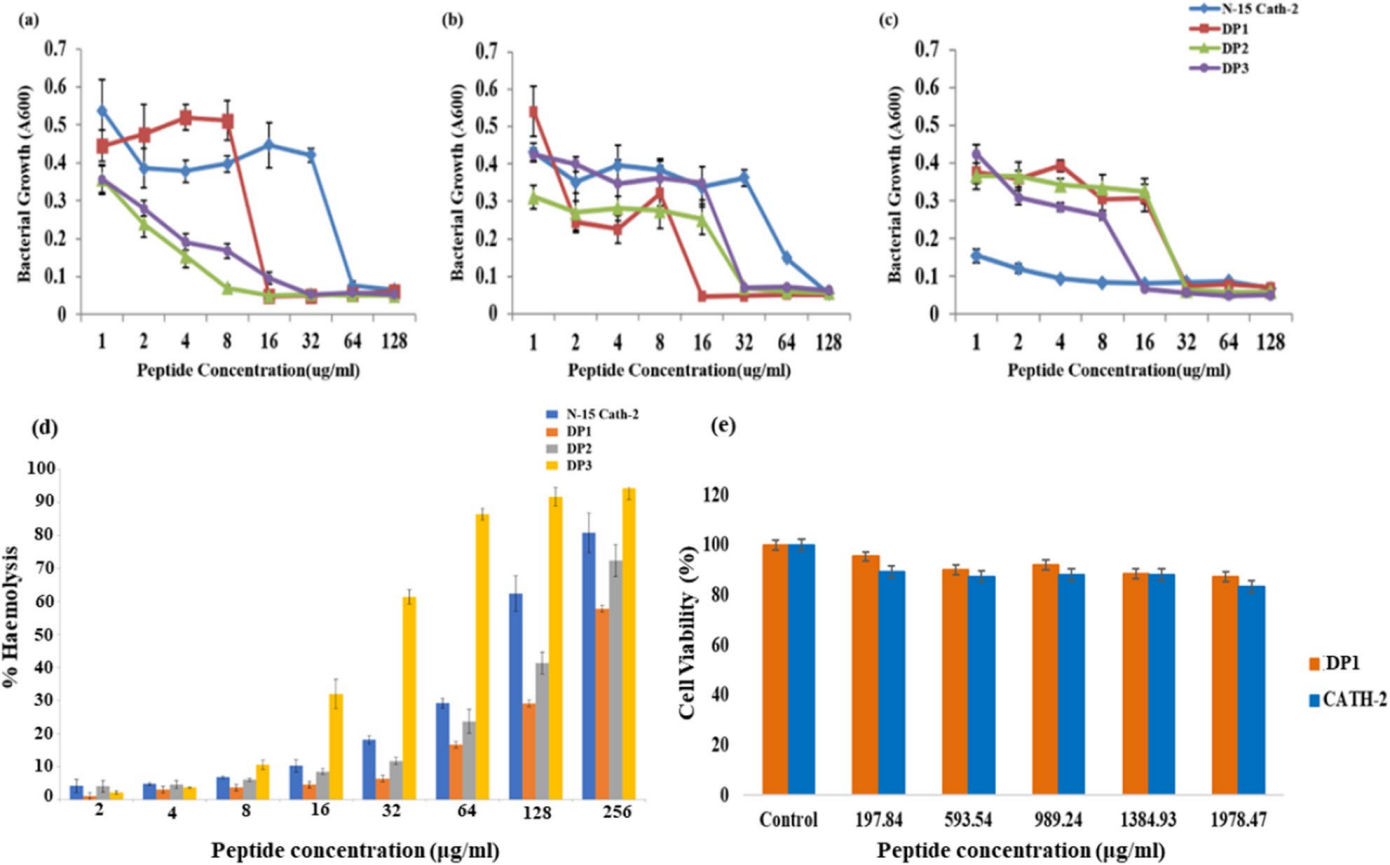
Antimicrobial (MIC) activities of peptides against *Escherichia coli* (**a**),* Salmonella typhimurium* (**b**) and *Staphylococcus aureus* (**c**); Haemolysis dose response curves obtained after incubation of 10% RBC suspension with peptide serial dilutions for 1 h at 37 °C (**d**); Murine macrophages were incubated with five different concentrations of DP1 and N-15 CATH-2 and percentage cell viability was analysed using MTT assay (**e**). Each bar denotes the mean ± SD of three independent experiment.

The effect of amino acid substitution on antimicrobial activity of the designed peptides was studied using microbroth dilution method against Gram-positive and Gram-negative microorganisms (Fig. 6a–c). Peptides were incubated with the microorganisms in a concentration ranging from 1 to 128 μg/ml. It was observed that all the peptides DP1, DP2 and DP3 inhibited the growth of microorganisms in a dose-dependent manner. The peptides affected the survival of all the tested bacterial strains whereas, N-15 CATH-2 displayed no antimicrobial activity against *Staphylococcus aureus* (*S. aureus*) even at a concentration of 128 μg/ml (Table 2). Due to the highly hydrophobic nature of DP3, the peptide is poorly soluble in aqueous environment, resulting in unstructured conformation and a reduced antimicrobial activity, whereas DP1, being soluble in aqueous environment, attains a helical structure and displays potent antimicrobial activity. Further, the difference in MIC values of the AMPs against Gram-positive and Gram-negative bacteria, can be explained by the difference in the membrane structure of both groups, with the Gram-negative bacteria being capable to restrict the diffusion of hydrophobic compounds through its Lipopolysaccharide envelope. The distinct protein and lipid composition of bacterial membrane, the length and charge of peptide chain also accounts for the difference in their MIC values. Consequently, different AMP exhibit different mechanism of action against Gram-positive and Gram-negative bacteria, hence giving rise to varying MIC values as seen in Table 2.

**Table 2:** MIC and HC50 activities of peptides against bacterial strains and murine red blood cells (RBCs).

Peptides	MIC in μg/mL			GM of MIC	HC_50_ in μg/mL	Therapeutic index (HC_50_/GM of MIC)	Fold
	*Escherichia coli* O175:H7	*Salmonella typhimurium* NCTC 74	*Staphylococcus aureus* ATCC 9155				
N-15 Cath-2	64	8	≥ 128	19.95	97.76	2.4	10
DP1 (LLK)	16	32	16	10.2	216	10.7	4.46
DP2 (LLR)	8	32	32	10.08	145	6.7	2.8
DP3 (L3)	32	16	32	25.4	24.62	0.5	0.2

Further, the overall evaluation of antimicrobial activity against the bacterial strains was done by calculating geometric mean (GM) of the obtained MIC which showed greater activity of DP1 and DP2 as compared to N-15 CATH-2. The GM value of DP1 and DP2 i.e., 10 μM approx. was much lower as compared to that of the N-15 CATH-2 whereas GM value of DP3 was observed to be higher (Table 2). Though the GM values of DP1 and DP2 is similar but there was a large difference in their (50% haemolysis of RBC) HC_50_ values which in turn lead to distinctive therapeutic index values. DP1 with lowest MIC value and highest HC_50_ value draws in the maximum therapeutic index value of 10.7 with 4.5-fold improvement (Table 2) thereby indicating increased antimicrobial specificity. The antibacterial activity of the peptides can be predominantly attributed to their net positive charge and a delicate balance between the aliphatic character and hydrophobic content which facilitates interaction with negatively charged bacterial membrane leading to its destabilization.

### Haemolytic activity

Haemolytic concentration of the peptides was determined as a reflection of their toxicity towards erythrocytes. Haemolytic activity of the peptides in a dose-dependent manner is shown in Fig. 6d. In comparison to N-15 CATH-2, DP1 and DP2 exhibited low haemolysis whereas DP3 exhibited highest haemolysis. The HC_50_ values for DP1 and DP2 were 216 and 146 μg/ml, respectively. HC_50_ occurred at a higher concentration in comparison with the MIC values thereby indicating therapeutic potential of peptides (Table 2). It should be noted that increased amphipathicity along with greater hydrophobicity are responsible for higher haemolytic values^42^. Since DP3 is highly hydrophobic as indicated by the hydropathy index it is poorly soluble in aqueous environment, therefore, increased haemolytic activity is obvious since, the peptide rapidly induces erythrocyte membrane aggregation and ruffling^43^.

### Cytotoxicity assay

The cytotoxicity of peptides against normal cells (murine macrophages) were determined using colorimetric MTT (3-(4-5-dimethylthiazol-1-yl)-2,5-diphenyltetrazolium bromide) assay. The percentage cell viability was expressed as the function of peptide concentration with reference to the negative control, which was regarded as 100%. As shown in Fig. 6e, at a concentration up to 1978.47 μg/mL, the cell viability was not affected considerably on treatment with DP1 (≈ 90% of the cells were viable). Also, DP1 was found to be less toxic to live cells than N-15 CATH-2. Further, our results also showed that cell viability decreased upon gradual increase in peptides concentration which leads to the conclusion that the cytotoxicity of cells towards the peptides follows a dose-depended behaviour i.e., higher inhibition of cells was observed with higher peptide concentrations. Cytotoxicity assay results show that DP1 represents an improvised version of N-15 CATH-2, validating it to be less cytotoxic when tested against murine macrophages.

## Methods

### Materials

Phospholipids (DMPC and DMPG) were purchased from Avanti Polar Lipids, Inc. TFE was purchased from Sigma-Aldrich. The peptides were synthesized commercially by GL Biochem (Shanghai, China) using solid phase Fmoc chemistry and purified to > 95% purity using reverse phase HPLC. The lyophilized peptide was stored at − 20 °C until use. Here, we have also used our patented peptide (DP1) for structural and functional studies. The study protocols were approved by the Research Monitoring Committee, Panjab University and also, Institutional Biosafety Committee, Panjab University, Chandigarh, India (IBSC/PU/2019/154-156).

### Animals

The Balb/c mice used in the study (30 ± 2 g) were obtained from the Central Animal House, Panjab University, Chandigarh. All the experiments involving animals were carried out in accordance with the guidelines and regulations of the Institutional Animal Ethics Committee, Panjab University, Chandigarh, India (PU/45/99/CPCSEA/IAEC/2019/375) and “The ARRIVE Guidelines”^44^. All efforts were made to minimize the animal suffering and the number of animals used. The animals were housed under a 12 h light/dark cycle in a temperature and humidity-controlled facility. Food and water were made available ad libitum.

### MD simulations

The peptides i.e., N-15 CATH-2 and the designed peptide analogs (DP1-5) were prepared using UCSF Chimera software^45^ for three starting conformations (A, B and C) with distinct backbone dihedral angles (*φ*, *ψ*) of (180°, 180°), (− 57°, − 47°) and (− 139°, 135°) corresponding to linear, alpha-helical, and beta-sheet structure, respectively. All molecular dynamics (MD) simulations were performed by using GROMACS (version 4.5.5) software package^46^ with a constant integration time step of 2 fs. Two different solvents were used, water (spc216 model)^47^ and DMSO^48^. The force field chosen for generating the coordinate and topology was G43a1force field^49^. The systems were rendered neutral by replacing solvent molecules by Chloride ions. The steric conflicts between solvent and model peptides were removed by subjecting the system to energy minimization using steepest descent method with the convergence value (emtol) of 1000 kJ mol^−1^ nm^−1^. In order to allow equilibration of solvent around peptides, the position of all residues was restrained for 20 ps at the desired temperature. MD simulations were performed for 20 ns in an NVT ensemble at a constant temperature of 300 K in simple cubic periodic box. Temperature and pressure were controlled through weak coupling to a bath of constant temperature^50^ using a coupling time constant (τ_p_) of 0.1 ps and 0.5 ps, respectively and a reference temperature (T_ο_) of 300 K and pressure of 1 Bar.

### MD simulations of peptide-micelle systems in implicit water

Initial micelle structure of Sodium dodecyl sulphate (SDS; 60 molecules) was built through micelle maker^51^ and Dodecyl-phosphocholine (DPC; 65 molecules) was downloaded from the Tieleman Laboratory following link http://wcm.ucalgary.ca/tieleman/downloads^52^. The anionic SDS micelles mimic a prokaryotic membrane environment and the zwitterionic DPC micelles mimic a eukaryotic environment^34^. Force field parameters of the single unit of SDS and DPC were parametrized using antechamber and General Amber Force Field library^53,54^ in the amber 18 modules. Force field parameters for the peptide, ff14SB force field library were used^55^. Five systems (SDS micelle with water, DPC micelle with water, SDS micelle-peptide with water, DPC micelle-peptide with water, and peptide with water) were prepared with peptide for the molecular dynamics simulation studies. For the DPC 0.15 M concentration was used and for SDS micelle charge neutralization, 56 Na + ions were added in the TIP3P water system^56^. All the simulation setup preparations were done using Amber 18 toolkit.

All the systems were minimized for 50,000 steps using conjugated gradient method. First equilibration was performed by heating the systems up to 300 K where position restraints were placed on the peptide while solvent and ions were moving. 2.5 ns NVT equilibration was used for the protein relaxation and 105 ns NPT was used for the equilibration where position restraints were removed and finally simulation production run was carried out for 100 ns. All the dynamics part and analysis were done in NAMD 2.13^57^ and VMD 1.9.3 version^58^.

### Circular dichroism spectroscopy

The secondary structure of the peptides was determined on a Jasco J-815 spectropolarimeter (Jasco, Tokyo, Japan) at constant temperature of 25 °C. Peptide samples were analyzed at 50 μM concentration in different mediums: (a) in 10 mM sodium phosphate buffer (SPB), (b) 25% trifluoroethanol (TFE) and 50% SPB. Spectra were recorded from 190 to 260 nm at a scanning speed of 10 nm min^−1^. TFE is a secondary structure-inducing material that mimics the membrane environment^59–61^. Since a peptide is unstructured and flexible in solution, it becomes increasingly structured at increasing TFE concentrations, hence TFE was used as a membrane-mimetic and stabilizing material for CD spectroscopic analysis^62^. The obtained CD signal spectra were then converted to mean residue ellipticity (θ) using the following formula:

1 $${\uptheta } = \frac{{\left( {{\text{obs}} \times 1000} \right)}}{{\left( {{\text{c}} \times {\text{l}} \times {\text{n}}} \right)}}$$

where, θ is the mean residue ellipticity (degrees square centimeter per decimole), obs is observed ellipticity (millidegrees), *c* is peptide concentration (millimoles), *l* is path length (millimeters), and *n* is number of amino acids. The percent helicity of the peptides was determined from the mean residue helicity at 222 nm with values of 0 and − 40,000 (1—2.5/n) deg cm^2^ dmol^−1^ per amino acid residue for 0 and 100% helicity^63^. The backgrounds were subtracted before the normalisation to MRE.

### Differential scanning calorimetry

Binding of drugs, peptides, and proteins to multilamellar vesicles (MLVs) can strongly perturb their structural integrity and promote changes in the thermodynamic parameters such as the melting temperature (T_m_), the calorimetric enthalpy change of the transition (ΔH) and transition cooperativity, hence significantly affecting their thermotropic phase behavior^64,65^. MLVs composed of zwitterionic phospholipid DMPC (1,2-dimyristoyl-sn-glycero-3-phosphocholine) and a mixture of DMPC/DMPG {1,2-dimyristoyl-sn-glycero-3-(phospho-rac-1-glycerol)} have been widely reported as models for mammalian and bacterial membranes, respectively^5,35^. Therefore, in this study, the changes in the organization and phase behavior of different MLVs in the absence and presence of peptides were investigated by DSC. The calorimetric enthalpy (∆H) was calculated by integrating the area under the transition peak obtained in DSC thermogram.

2 $$\Delta {\text{H}} = {\text{KA}}$$

where, K is the calorimetric constant, and A is the area under the curve. Cooperativity was calculated by the ratio of ∆H_vH_/∆H, where ∆H_vH_ is the van’t Hoff enthalpy given by^66^,

3 $$\left( {{\text{dlnK}}/{\text{dT}}} \right) \, = \, \Delta {\text{H}}_{{{\text{vH}}}} /{\text{RT}}^{2}$$

where, K is the equilibrium constant of the process, and T is the absolute temperature. DSC was recorded on a PerkinElmer Differential Scanning Colorimeter, DSC 8000 model, and the samples were heated from 5 to 50 °C at a heating rate of 2 °C/min under a nitrogen atmosphere^67^.

### Antimicrobial assay

Minimum inhibitory concentration (MIC) values of peptides were determined by a standard broth microdilution method^68^ in Mueller–Hinton broth (MHB). . It is the lowest antimicrobial concentration that will inhibit the visible growth of a microorganism after overnight incubation^69^. Bacterial cells in mid-log phase were diluted to keep inoculums concentration to nearly 1 × 10^5^ CFU/ml. Peptides dissolved in 0.01% acetic acid in PBS were serially diluted and added to each well of the 96-well plates in a volume of 50 µl, followed by 50 µl of inoculum. The plates were incubated at 37 °C for 18 to 24 h. After incubation, absorbance of each well was recorded at 600 nm. The MIC was taken as the lowest concentration of the peptide that prevented visible turbidity. MIC analysis was done on bacterial strains including Gram-negative strains of *Escherichia coli* and *Salmonella typhimurium* and Gram-positive strain of *Staphylococcus aureus* ATCC 9144 provided by Department of Microbiology, Panjab University, Chandigarh. The experiments were carried out in three independent triplicates. Geometric mean (GM) was calculated from the MIC values of the all the bacterial strains mentioned in Table 2, using the following equation:

4 $$\mathrm{GM}=n\sqrt{MIC1. MIC2.MIC3}$$

where, n = number of MIC values.

### Haemolysis assay

The haemolytic activity of the peptides was determined by the released haemoglobin from suspensions of fresh murine erythrocytes as absorbance at 540 nm^70^. Ethylenediamine tetraacetic acid (EDTA) anti‐coagulated blood was centrifuged for 10 min at 2500 rpm (4 °C) to sediment the red blood cells (RBCs). The buffy coat and serum fraction was removed, and pelleted RBCs were washed with PBS. The RBC suspension was diluted to concentration of 10% in PBS. Serial peptide dilutions were mixed with an equal volume of 10% RBC suspension and incubated for 1 h at 37 °C. Supernatants were collected after 10 min centrifugation at 2500 rpm (4 °C) and absorbance was measured at 540 nm. The control samples for 0% and 100% haemolysis consisted of RBCs in PBS (negative control) only and in 0.1% Triton X-100 (positive control), respectively. The percentage of haemolysis was calculated according to the following equation:

5 $${\text{Percent Haemolysis}} \,\left( \% \right) = \frac{{\left( {{\text{sample abs at}}\,540 {\text{nm}} - {\text{negative control abs at}}\,540{\text{ nm}}} \right)}}{{\left( {{\text{positive control abs at}}\,540 {\text{nm}} - {\text{negative control abs at}}\,540\,{\text{nm}}} \right)}} \times 100$$

Statistical analyses were performed using GraphPad Prism software v.5. Results are compared using two-way ANOVA (Bonferroni method) and expressed as the mean ± standard deviation of three independent observations. Obtained results were validated and were considered significant for p ≤ 0.05.

### Isolation of peritoneal macrophages

Peritoneal macrophages were isolated from Balb/c mice by a previously described protocol^71,72^. Wherein, thioglycolate broth (2.5 ml i.p/mouse) was injected 4 days in advance to have a better yield of macrophages. Further, the obtained macrophages were washed and centrifuged with RPMI-1640 medium at 400 g for 10 min. Yield of 10^6^ macrophages was obtained. Cell viability of the macrophages was tested by conducting trypan blue staining (0.2%). Murine macrophages were maintained in RPMI-1640 with 10% foetal bovine serum (FBS) and 1% penicillin–streptomycin for further use.

### Cytotoxicity assay

The colorimetric 3-(4,5-dimethylthiazol-2yl)-2,5-diphenyl tetrazolium bromide (MTT) assay was used to determine the cytotoxicity of N-15 CATH-2 and DP1 by assessing the metabolic activity of treated cells in comparison to the untreated control^73^. The cells (10^7^ cells/mL) were plated and incubated in the humidified atmosphere containing 5% CO_2_ at 37 °C for 24 h. After incubation, the media was replaced with 100 μl of fresh RPMI-1640 containing various concentrations of peptides and were left for 24 h in the same growth conditions. Followed by this, 20 μl of MTT reagent (5 mg/mL) was added to each well and further incubated at 37 °C for 4 h for exponential growth of cells. The viable cells metabolize MTT to formazan crystals, hence, 100 µl DMSO per well was added to dissolve the formazan crystals followed by removing media from each well. The absorbance was read spectrophotometrically at 595 nm using an ELISA plate reader to estimate the number of viable cells. Once cells die, they lose the potential to change yellow MTT into purple formazan crystals; hence colour formation plays the role of a convenient and effective marker for only viable cells. A statistical analysis was performed using Kruskal Wallis Test (*p* ≤ 0.05).

## Conclusion

Inspired by the promising results of several peptide-based drugs, various design strategies and approaches have been employed to explore naturally occurring AMPs as therapeutic agents against different pathogens. Moreover, the emergence of new strains of pathogenic organisms and even more resistant ones have urged the need for novel antimicrobials with appropriate pharmacokinetic and toxicological properties. This need of the hour has been addressed and worked upon in this study. Here we have designed five analogs of N-15 CATH-2 (DP1, DP2, DP3, DP4 and DP5). Based on the structural insights, DP1, DP2 and DP3 peptides were used for structural activity relationship studies. Our results have elucidated various properties of novel peptide DP1 such as low cytotoxicity, low haemolytic activity, fluidification of membrane, efficient antimicrobial activity, strong helical properties, stability in the membrane-mimicking environment and amphipathic helical secondary structure irrespective of the solvent. The amphipathic nature of the peptide arises due to its strong binding with core of the micelle as well as with the surface. Thus, it disorganizes the membrane structure and increases the water fluidity inside the micelle. These observations project the novel peptide as a suitable candidate to be further explored in various pharmacological paradigms.

## Supplementary Information

Supplementary Information.

## Data Availability

All relevant data are available from the corresponding author upon reasonable request. The source data generated during the current study has been included in this article and its Supplementary Information files S1.
