# Supplementary material for: Design, characterization and structure–function analysis of novel antimicrobial peptides based on the N-terminal CATH-2 fragment
Source: Sci Rep. 2022 Jul 14;12:12058. doi: 10.1038/s41598-022-16303-2 (PMC9283491; doi:10.1038/s41598-022-16303-2)
Supplement: Supplementary file 1 — Supplementary Information. [file 41598_2022_16303_MOESM1_ESM.docx]

**
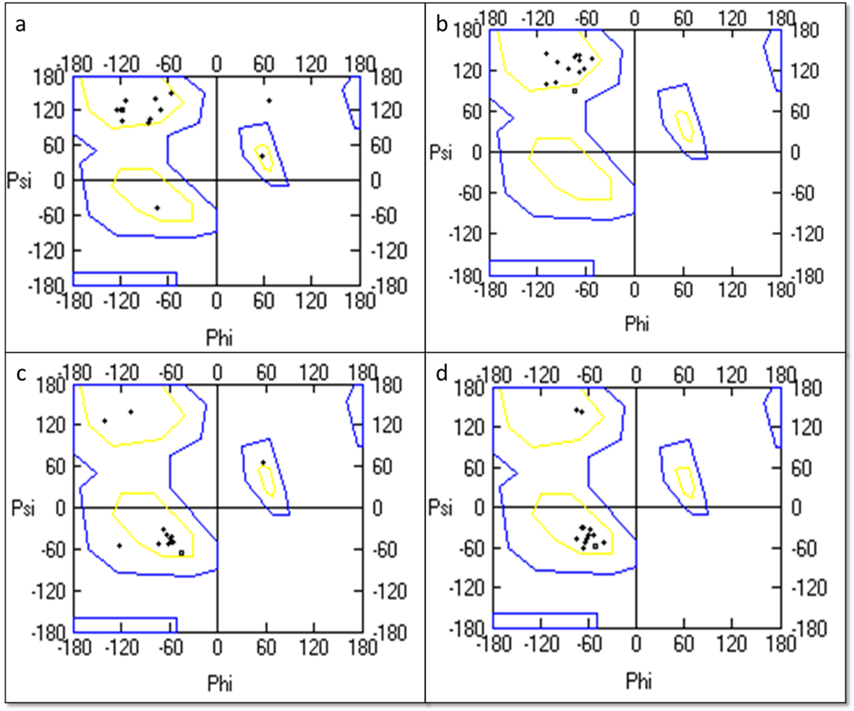
Supplementary material**

**Supplementary Figure 1: Ramachandran Plots showing the stereo chemically allowed combinations of the Φ, ψ values for CATH-2 (a, b) and DP1 (c, d) in water and DMSO respectively after 20ns simulations.**

**
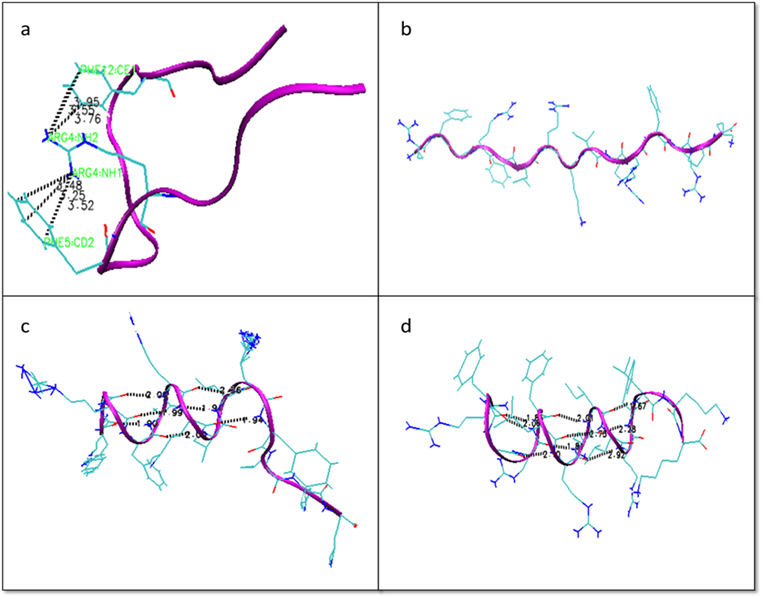
**

**Supplementary Figure 2:** Molecular views showing the stereo chemically allowed conformations for CATH-2 (**a**, **b**) and DP1 (**c**, **d**) in water and DMSO respectively after 20ns simulations.


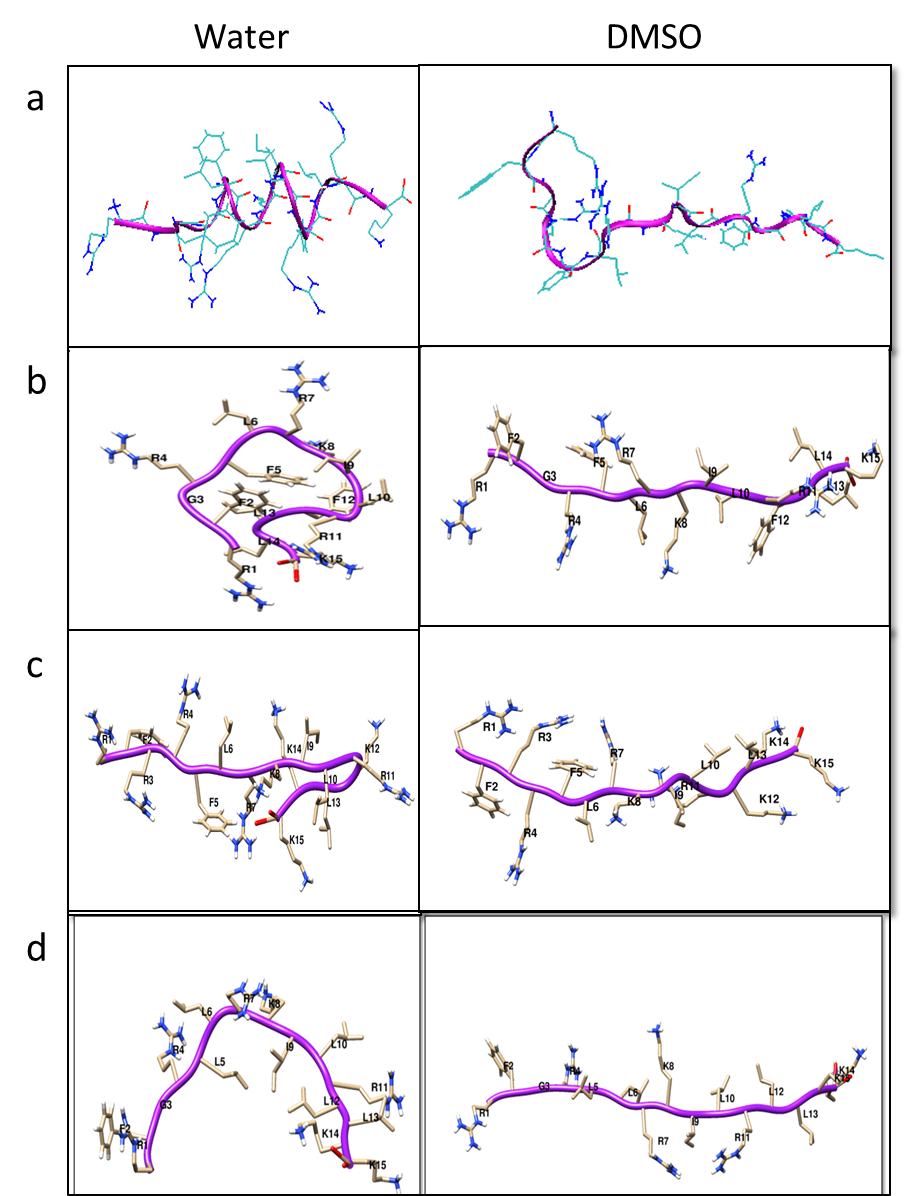


**Supplementary Figure 3: Molecular views of the average structure of energetically favourable conformations of DP2 (a), DP3 (b), DP4 (c) and DP5 (d) after 20ns simulation in water and DMSO.**


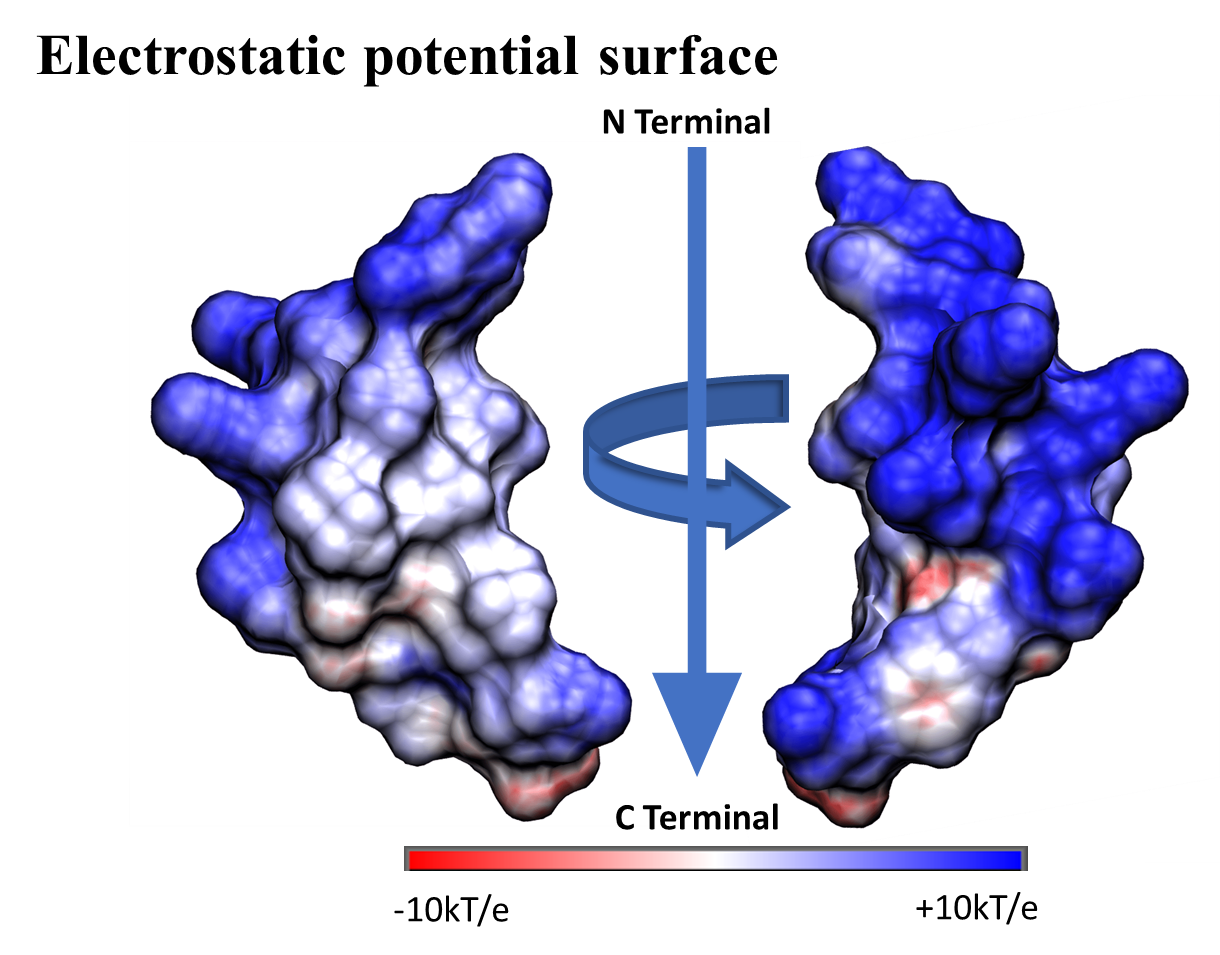


**Supplementary Figure 4: Electrostatic potential surface of the peptide calculated through PDB2PQR server.**

**
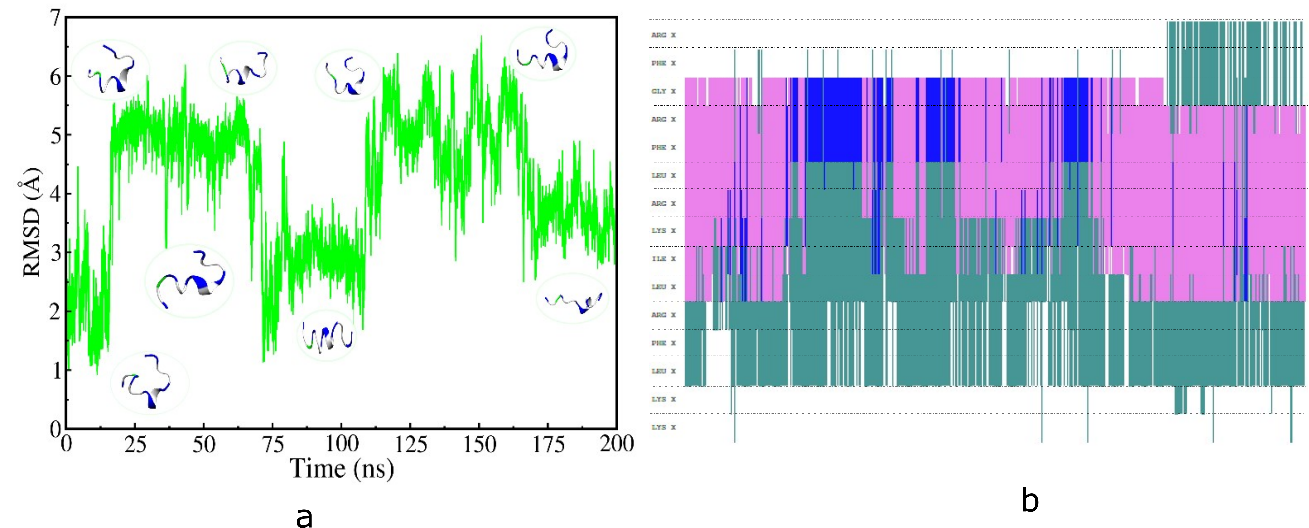
**

**Supplementary Figure 5. Peptide RMSD in the water where selected peptide confirmation is based on RMSD minimum and maximum (a). Secondary structure of the peptide molecule in the 100ns dynamics (b).**

**
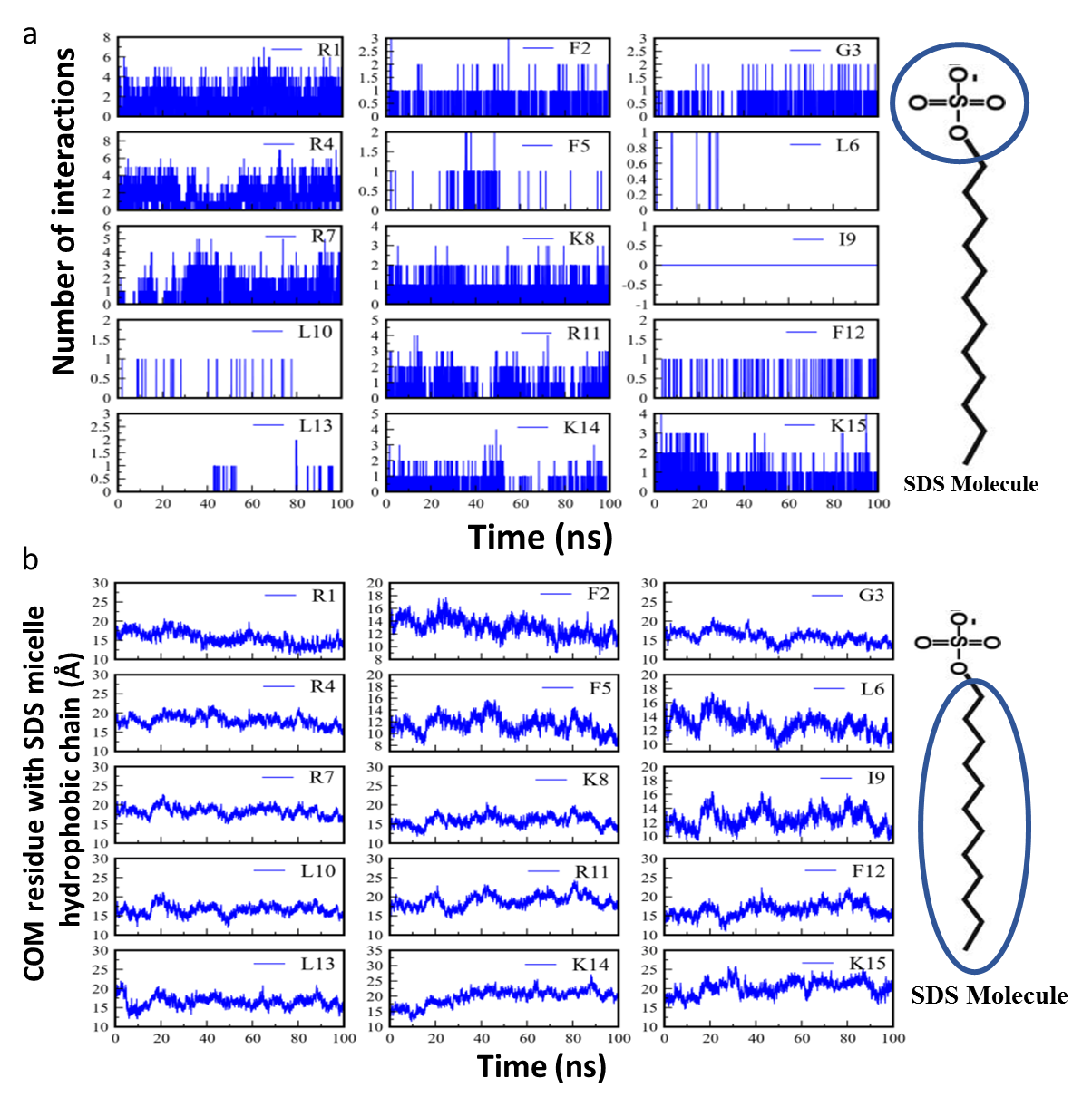
**

**Supplementary Figure 6: a) No. of H-bond interactions between each amino acid and SDS (oxygen and sulfur atoms) (a) and Distance between Center of mass (COM) of each residue and the Hydrophobic core (Heavy atoms) of the SDS micelle (b).**

**
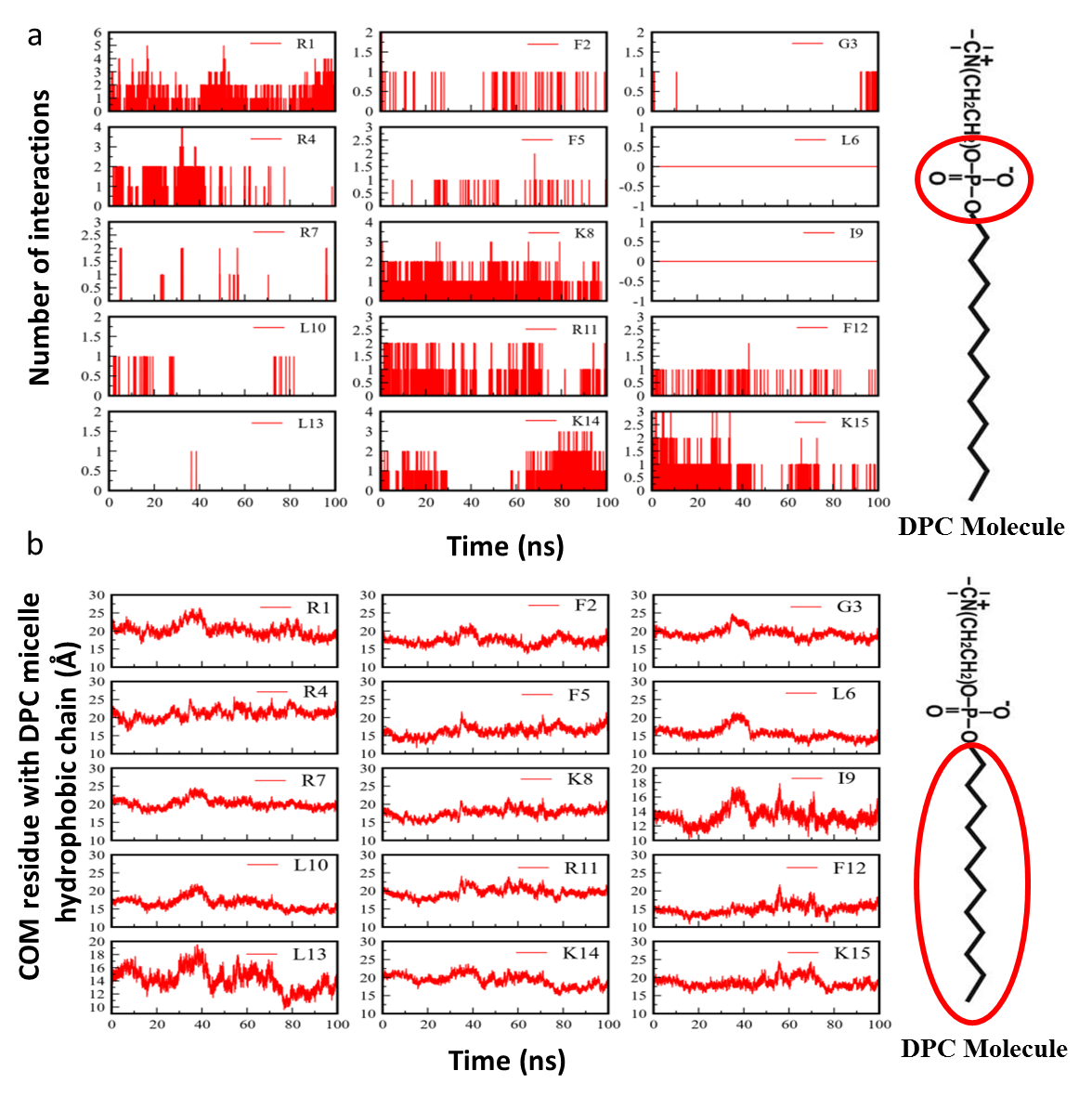
**

**Supplementary Figure 7: No. of H-bond interactions between each amino acid and DPC micelle (oxygen atoms) (a) and Distance between Center of mass (COM) of each residue and the Hydrophobic core (Heavy atoms) of the DPC micelle (b).**

**Supplementary Table 1: Conformational results of average structures of N1-15 CATH-2 peptide after 20ns MD simulations in water (A) and DMSO (B)**

**A**

**B**


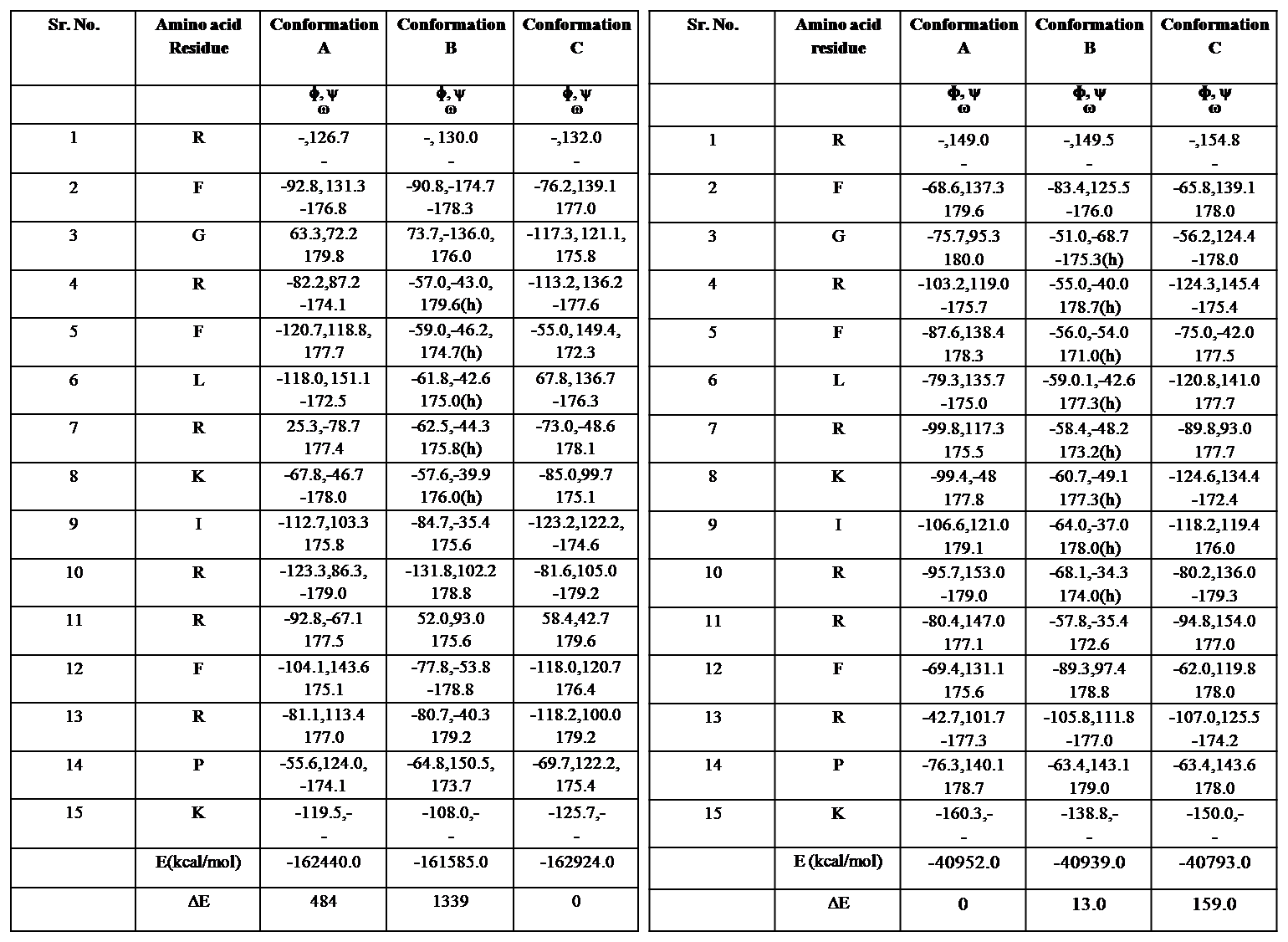


**Supplementary Table 2: Conformational results of average structures of DP1 after 20ns MD simulations in water (A) and DMSO (B)**


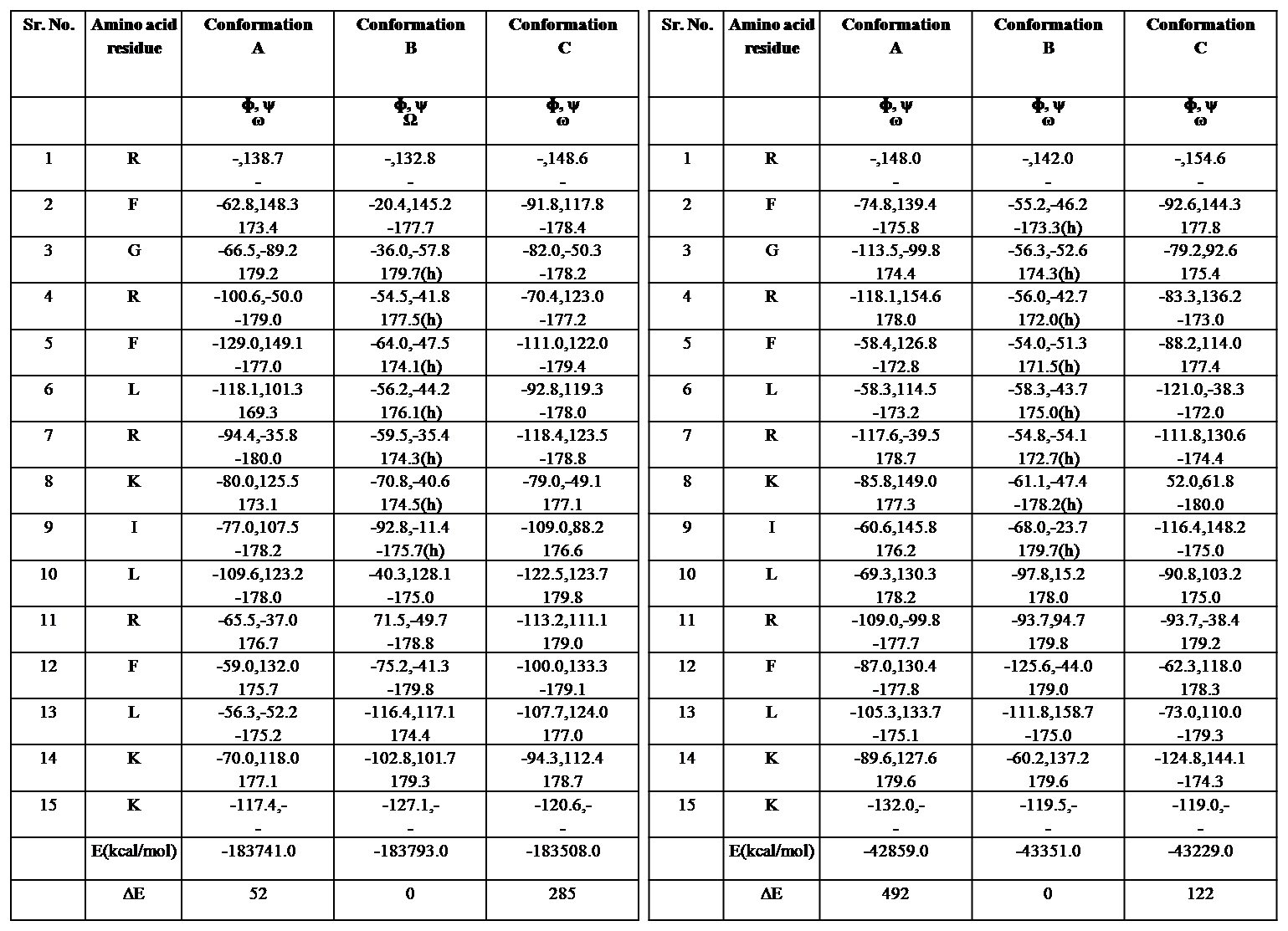
**A B**

**Supplementary Table 3: Conformational results of average structures of DP2 after 20ns MD simulations in water (A) and DMSO (B)**

**A B**

| **Sr. No.** | **Amino acid**  **Residue** | **Conformation A** | **Conformation B** | **Conformation C** | **Amino acid**  **residue** | **Conformation A** | **Conformation B** | **Conformation C** |
| --- | --- | --- | --- | --- | --- | --- | --- | --- |
|  |  | **ɸ, ψ**  **ω** | **ɸ, ψ**  **ω** | **ɸ, ψ**  **ω** |  | **ɸ, ψ**  **ω** | **ɸ, ψ**  **ω** | **ɸ, ψ**  **ω** |
| 1 | R | -, 138.7  - | -,134.7  - | -,144.6  - | R | -,148.0  - | -,151.7  - | -,138.0  - |
| 2 | F | -100.7,133.7  -178.5 | -108.5,86.5  179.8 | -125.2,124.7  -179.7 | F | -69.0,155.6  173.5 | -115.8,145.6  -178.6 | -98.9,-51.3  173.8 |
| 3 | G | 40.1,59.6  177.2 | -76.5,178.6  -177.8 | -92.6,-77.5  173.7 | G | 116.5,-121.0  -168.8 | -44.0,131.0  165.8 | 80.4,-171.7  178.0 |
| 4 | R | 59.7,87.7  -171.3 | -54.0,-48.0  -176.8 | -97.0,101.0  173.5 | R | -62.1,136.3  167.5 | -53.3,-54.7  -177.8 | -116.4,-57.5  178.8 |
| 5 | F | 68.0,-66.8  -175.7 | -59.0,-47.3  174.6 | -78.8,138.0  -179.1 | F | -63.0,139.7  -179.8 | -64.2,-33.3  179.5 | -127.0,-40.5  -174.0 |
| 6 | L | -137.5,148.0  -176.5 | -60.2,-41.1  174.5 | -84.0,105.7  175.0 | L | -58.8,139.0  179.0 | -64.0,-37.6  167.7 | -117.0,155.8  -171.6 |
| 7 | R | -64.0,-19.3  172.0 | -58.1,-46.5  172.3 | -71.0,118.0  -175.0 | R | -113.8,149.2  175.0 | -61.0,-45.5  176.4 | -88.2,-59.0  177.0 |
| 8 | K | -64.0,130.4  172.0 | -60.4,-58.8  175.0 | -82.0,-52.3  177.4 | K | 8.8,103.8  174.2 | -59.2,-60.0  178.5 | -84.4,134.2  -175.4 |
| 9 | I | -45.6,-38.2  -170.7 | -65.0,-49.6  -177.4 | -118.4,-83.4  174.6 | I | -73.5,130.0  -169.2 | -73.8,-38.7  -165.3 | -75.7,133.0  -179.0 |
| 10 | L | -62.2,-31.4  174.5 | -83.7,-49.7  -176.4 | -123.7,-49.0  -178.7 | L | -92.1,134.2  -165.0 | -113.6,138.2  -179.4 | -61.7,115.8  -177.7 |
| 11 | R | -99.4,-40.6  176.3 | -86.3,-51.3  -176.8 | -71.7,135.4  -174.2 | R | -114.3,95.2  -175.5 | -136.7,71.0  -174.3 | -114.2,141.3  179.0 |
| 12 | F | -50.7,113.2  -176.1 | -71.1,-53.2  179.5 | -103.6,108.1  176.0 | F | -94.7,95.7  -178.0 | -123.8,-20.5  -174.6 | -111.0,-50.2  -172.2 |
| 13 | L | 56.4,18.4  -177.8 | -75.5,-40.4  -179.7 | -104.3,104.0  -175.0 | L | -119.4,109.5  177.0 | -118.4,155.3  171.8 | 47.8,109.2  -174.0 |
| 14 | R | -101.0,-60.7  -178.0 | -91.0,124.0  179.2 | -94.3,129.3  179.2 | R | -67.7,98.6  175.0 | -118.6,-45.8  175.0 | -84.8,125.2  -174.0 |
| 15 | K | -125.7,-  - | -115.6,-  - | -137.0,-  - | K | -124.6,-  - | -174.6,-  - | -146.4,-  - |
|  | Etot | -185989.0 | -186304.0 | -184192.8 | Etot | -44437.6 | -44688.9 | -44736.5 |
|  | ∆E | 315 | 0 | 2111 | ∆E | 299 | 47.6 | 0 |

**Supplementary Table 4: Conformational results of average structures of DP3 after 20ns MD simulations in water (A) and DMSO (B)**

**A B**

| **Sr. No.** | **Amino acid**  **Residue** | **Conformation A** | **Conformation B** | **Conformation C** | **Amino acid**  **residue** | **Conformation A** | **Conformation B** | **Conformation C (beta sheet)** |
| --- | --- | --- | --- | --- | --- | --- | --- | --- |
|  |  | **ɸ, ψ**  **ω** | **ɸ, ψ**  **ω** | **ɸ, ψ**  **ω** |  | **ɸ, ψ**  **ω** | **ɸ, ψ**  **ω** | **ɸ, ψ**  **ω** |
| 1 | R | -,123.7  - | -,135.8  - | -,135.2  - | R | -,84.8  - | -, 142.0  - | -,160.0  - |
| 2 | F | -40.5,84.0 | -100.7,112.0  -179.6 | -89.4,135.0  178.3 | F | -64.1,129.0  -172.2 | -66.0,-71.6  167.6 | -103.1,135.1  178.2 |
| 3 | G | -59.0,100.0 | -67.6,162.8  -178.4 | -81.7,-86.7  -178.7 | G | -168.0,164.0  -168.7 | 144.3,140.6  178.6 | 173.6,-93.3  -177.8 |
| 4 | R | -99.0,72.2 | -58.6,-51.0  -176.7 | -88.5,136.2  -176.8 | R | -92.0,110.7  -171.0 | -94.6,108.1  176.2 | -131.6,122.0  -167.3 |
| 5 | F | -61.0,112.0 | -61.8,-43.8  176.2 | -98.5,140.0  -177.0 | F | -145.4,153.2  173.5 | -111.3,-66.8  168.0 | -175.0,147.0  175.7 |
| 6 | L | -71.3,-35.8 | -56.1,-44.7  172.6 | -81.0,100.0  178.5 | L | -58.0,98.3  -170.5 | -45.8,-45.8  176.7 | -153.0,-41.3  -172.4 |
| 7 | R | -33.0,-97.6 | -58.7,-45.8  173.1 | -131.3,103.2  177.0 | R | -71.7,159.6  -179.8 | -77.0,-35.7  -179.0 | -123.2,149.5  166.8 |
| 8 | K | -91.2,104.0 | -58.5,-53.8  176.0 | -81.8,99.8  -176.0 | K | -61.8,102.2  -172.0 | -68.0,-45.5  171.0 | -54.6,162.6  165.7 |
| 9 | I | -66.8,97.0 | -64.1,-36.8  179.2 | -137.5,115.8  179.4 | I | -69.8,194.8  177.7 | -74.0,-36.8  -179.5 | -134.1,176.0  -177.6 |
| 10 | L | -58.0,113.0 | -62.0,-43.0  172.2 | 29.7,89.5  -179.8 | L | -73.8,120.2  -170.6 | -77.6,-37.5  173.7 | -115.6,134.7  178.0 |
| 11 | R | -64.0,122.0 | -63.2,-38.8  173.8 | 68.7,-32.3  -174.0 | R | -90.0,130.4  -170.2 | -105.0,145.7  -171.6 | -73.6,128.3  162.2 |
| 12 | F | -61.4,84.2 | -64.8,-42.3  173.8 | -87.4,138.6  -179.5 | F | -63.8,133.0  177.8 | -63.6,124.2  168.0 | -138.4,149.2  -178.5 |
| 13 | L | -84.0,131.0 | -68.1,-43.4  176.0 | -110.9,-43.5  -179.5 | L | -100.0,136.1  -162.8 | -68.8,108.6  -172.0 | -85.1,-45.0  -177.4 |
| 14 | L | -72.3,84.5 | -93.0,112.5  -179.7 | -105.8,137.0  -177.6 | L | -109.4,26.6  179.4 | -88.3,70.7  167.0 | -63.0,89.6  -179.2 |
| 15 | K | -146.4,-  - | -115.7,-  - | -126.0,-  - | K | -108.0,-  - | -137.0,-  - | -132.0,-  - |
|  | Etot | -185134.3 | -183703.7 | -186769.0 | Etot | -44860.0 | -44588.3 | -45621.2 |
|  | ∆E | 1635 | 3065 | 0 | ∆E | 761 | 1033 | 0 |

**Supplementary Table 5: Conformational results of average structures of DP4 after 20ns MD simulations in water (A) and DMSO (B)**

**A B**

| **Sr. No.** | **Amino acid**  **Residue** | **Conformation A (linear) in water** | **Conformation B (alpha-helix) in water** | **Conformation C (beta sheet) in water** | **Amino acid**  **Residue** | **Conformation A (linear) in DMSO** | **Conformation B (alpha-helix) in DMSO** | **Conformation C (beta sheet) in DMSO** |
| --- | --- | --- | --- | --- | --- | --- | --- | --- |
|  |  | **ɸ, ψ**  **ω** | **ɸ, ψ**  **ω** | **ɸ, ψ**  **ω** |  | **ɸ, ψ**  **ω** | **ɸ, ψ**  **ω** | **ɸ, ψ**  **ω** |
| 1 | R | -,141.6  - | -,146.3  - | -,138.5  - | R | -,106.5  - | -,145.3  - | -,134.4  - |
| 2 | F | -64.1,143.4  -179.0 | -85.6,138.7  -177.8 | -94.3,132.3  -179.2 | F | -145.6,129.0  178.5 | -56.4,-53.1  -177.2 | -135.0,148.6  177.0 |
| 3 | R | -86.8,131.4  176.7 | -49.8,-46.2  -176.0 | -105.2,122.0  178.0 | R | -74.2,115.5  -179.6 | -65.4,-40.6  178.7 | -142.6,102.0  177.3 |
| 4 | R | -68.6,113.5  -176.3 | -61.7,-44.6  177.0 | -103.1,128.8  -178.8 | R | -106.0,101.2  -173.5 | -55.3,-49.3  171.5 | -103.3,87.4  -178.6 |
| 5 | F | -122.4,140.0  179.0 | -58.0,-47.2  171.6 | -61.0,136.0  179.0 | F | -117.3,-45.3  179.2 | -58.5,-45.8  173.0 | -124.5,139.8  -177.8 |
| 6 | L | -71.3,-41.3  176.8 | -57.3,-39.0  176.4 | -114.2,126.6  -179.2 | L | -70.8,-45.6  173.3 | -57.2,-45.8  174.7 | -130.5,82.2  171.6 |
| 7 | R | -95.2,125.5  175.4 | -60.7,-54.2  171.0 | -74.6,-41.3  -177.7 | R | -74.2,115.5  175.7 | -59.4,-40.6  173.0 | -92.7,100.2  -179.8 |
| 8 | K | -96.7,122.0  -175.2 | -53.6,-41.0  178.6 | -63.5,115.3  175.3 | K | -120.3,146.2  -169.5 | -62.0,-44.6  175.0 | -116.5,-47.7  179.5 |
| 9 | I | -76.0,105.6  178.8 | -81.6,-43.7  176.0 | 49.3,55.5  -167.0 | I | -86.0,146.2  172.7 | -83.0,-30.6  -179.5 | -117.6,157.0  -177.4 |
| 10 | L | -114.7,93.0  176.4 | -129.8,-49.2  -172.5 | 8.0,92.8  -175.4 | L | -58.0,129.6  -176.4 | -127.2,109.7  -179.8 | -83.4,132.0  177.2 |
| 11 | R | 61.5,-85.0  -173.2 | -76.2,164.0  -167.7 | -106.7,108.7  -179.3 | R | -70.1,101.2  177.0 | -120.4,143.2  -177.8 | -79.5,158.4  171.4 |
| 12 | K | -110.0,-26.0  173.8 | -52.0,132.4  179.3 | -80.8,90.0  -174.0 | K | -111.0,-54.6  174.5 | -78.4,-40.0  -180.0 | -57.6,124.4  176.6 |
| 13 | L | -92.7,137.4  -178.6 | 58.0,93.7  -177.3 | -113.8,108.6  177.3 | L | -134.0,85.3  175.5 | -51.6,81.0  -179.6 | -78.2,149.5  -175.6 |
| 14 | K | -103.3,113.0  174.2 | -81.0,20.4  -174.3 | -102.0,-99.0  -174.7 | K | -115.3,137.4  -175.3 | -96.0,150.8  -175.7 | -76.6,129.0  180.0 |
| 15 | K | -111.3,-  - | -117.0,-  - | -121.0,-  - | K | -118.2,-  - | -113.3,-  - | -94.7,-  - |
|  | Etot | -186038.7 | -186052.2 | -185560.0 | Etot | -43149.0 | -45241.8 | -45657.4 |
|  | ∆E | 13.5 | 0 | 492.2 | ∆E | 2508 | 416 | 0 |

**Supplementary Table 6: Conformational results of average structures of DP5 after 20ns MD simulations in water (A) and DMSO (B)**

**A B**

| **Sr. No.** | **Amino acid**  **Residue** | **Conformation A** | **Conformation B** | **Conformation C** | **Amino acid**  **Residue** | **Conformation A** | **Conformation B** | **Conformation C** |
| --- | --- | --- | --- | --- | --- | --- | --- | --- |
|  |  | **ɸ, ψ**  **ω** | **ɸ, ψ**  **ω** | **ɸ, ψ**  **ω** |  | **ɸ, ψ**  **ω** | **ɸ, ψ**  **ω** | **ɸ, ψ**  **ω** |
| 1 | R | -,144.4  - | -,146.0  - | -,147.0  - | R | -,106.5  - | -,145.3  - | -,134.4  - |
| 2 | F | -88.0,146.4  175.5 | -74.3,115.0 | -111.8,151.0 | F | -145.6,129.0  178.5 | -56.4,-53.1  -177.2 | -135.0,148.6  177.0 |
| 3 | G | -170.6,119.6  176.0 | -75.2,167.4 | -108.7,-148.2 | G | -74.2,115.5  -179.6 | -65.4,-40.6  178.7 | -142.6,102.0  177.3 |
| 4 | R | -113.0,97.7  -177.0 | -55.4,-54.2 | -74.7,121.6 | R | -106.0,101.2  -173.5 | -55.3,-49.3  171.5 | -103.3,87.4  -178.6 |
| 5 | L | -103.6,-41.1  171.3 | -62.0,-43.5 | -87.0,67.0 | L | -117.3,-45.3  179.2 | -58.5,-45.8  173.0 | -124.5,139.8  -177.8 |
| 6 | L | -88.3,100.0  165.0 | -59.7,-43.4 | -62.7,115.3 | L | -70.8,-45.6  173.3 | -57.2,-45.8  174.7 | -130.5,82.2  171.6 |
| 7 | R | -78.0,68.4  -171.8 | -60.0,-42.8 | 63.3,-33.3 | R | -74.2,115.5  175.7 | -59.4,-40.6  173.0 | -92.7,100.2  -179.8 |
| 8 | K | -136.0,151.4  178.6 | -63.8,-49.6 | -108.5,127.6 | K | -120.3,146.2  -169.5 | -62.0,-44.6  175.0 | -116.5,-47.7  179.5 |
| 9 | I | -70.0,99.6  174.6 | -84.0,-39.0 | -80.3,33.5 | I | -86.0,146.2  172.7 | -83.0,-30.6  -179.5 | -117.6,157.0  -177.4 |
| 10 | L | -126.0,138.0  -174.2 | -89.8,-41.0 | -101.2,134.0 | L | -58.0,129.6  -176.4 | -127.2,109.7  -179.8 | -83.4,132.0  177.2 |
| 11 | R | -64.0,-38.6  173.3 | -103.8,101.7 | -76.0,48.0 | R | -70.1,101.2  177.0 | -120.4,143.2  -177.8 | -79.5,158.4  171.4 |
| 12 | L | -66.8,111.5  167.0 | -99.0,-53.8 | -108.3,-39.3 | L | -111.0,-54.6  174.5 | -78.4,-40.0  -180.0 | -57.6,124.4  176.6 |
| 13 | L | -68.0,-39.0  -172.7 | -93.0,-54.5 | -72.3,114.6 | L | -134.0,85.3  175.5 | -51.6,81.0  -179.6 | -78.2,149.5  -175.6 |
| 14 | K | -109.0,105.4  176.2 | -100.7,109.3 | -115.0,89.6 | K | -115.3,137.4  -175.3 | -96.0,150.8  -175.7 | -76.6,129.0  180.0 |
| 15 | K | -167.0,-  - | -112.4,-  - | -133.4,-  - | K | -118.2,-  - | -113.3,-  - | -94.7,-  - |
|  | Etot | -185922.3 | -176970.2  (unstable) | -184985.7 | Etot | -45493.0 | -45274.5 | -44273.0 |
|  | ∆E | 0 | 8952 | 936.6 | ∆E | 0 | 218.5 | 1220 |

**Supplementary Table 7: The effect of peptides on the thermotropic behavior of DMPC and DMPC/DMPG MLVs.**

|  |  | ΔT _m_ (˚C) | ΔH (cal/mol/˚C) |
| --- | --- | --- | --- |
| **DMPC** |  |  | 5749±71.8 |
| P1/DMPC | 1:100 | -0.3 | 5637±92.9 |
| P1/DMPC | 1:50 | -0.15 | 4992±89.8 |
| P1/DMPC | 1:10 | -0.17 | 4823±85.2 |
| N-15 Cath-2 /DMPC | 1:50 | -2.82 | 5696±52.2 |
| **DMPC/DMPG** |  |  | 7014±38.5 |
| P1/DMPC/DMPG | 1:100 | 0.04 | 4202±89.2 |
| P1/DMPC/DMPG | 1:50 | 0.18 | 3209±42.0 |
| P1/DMPC/DMPG | 1:10 | -5.74 | 1194±17.4 |
| N-15 Cath-2 /DMPC /DMPG | 1:50 | 0.06 | 4615±60.2 |
